# Radiofrequency to Microwave Coherent Manipulation of an Organometallic Electronic Spin Qubit Coupled to a Nuclear Qudit

**DOI:** 10.1021/acs.inorgchem.1c01267

**Published:** 2021-07-15

**Authors:** Matteo Atzori, Elena Garlatti, Giuseppe Allodi, Simone Chicco, Alessandro Chiesa, Andrea Albino, Roberto De Renzi, Enrico Salvadori, Mario Chiesa, Stefano Carretta, Lorenzo Sorace

**Affiliations:** † Dipartimento di Chimica “Ugo Schiff” e UdR INSTM, Università degli Studi di Firenze, Via della Lastruccia 3, I-50019 Sesto Fiorentino (Firenze), Italy; ‡ Laboratoire National des Champs Magnétiques Intenses (LNCMI), Univ. Grenoble Alpes, INSA Toulouse, Univ. Toulouse Paul Sabatier, EMFL, CNRS, F-38043 Grenoble, France; § Università di Parma, Dipartimento di Scienze Matematiche, Fisiche e Informatiche, I-43124 Parma, Italy; ¶ UdR Parma, INSTM, Parma, Italy; ∥ Dipartimento di Chimica e NIS Centre, Università di Torino, Via P. Giuria 7, I-10125 Torino, Italy

## Abstract

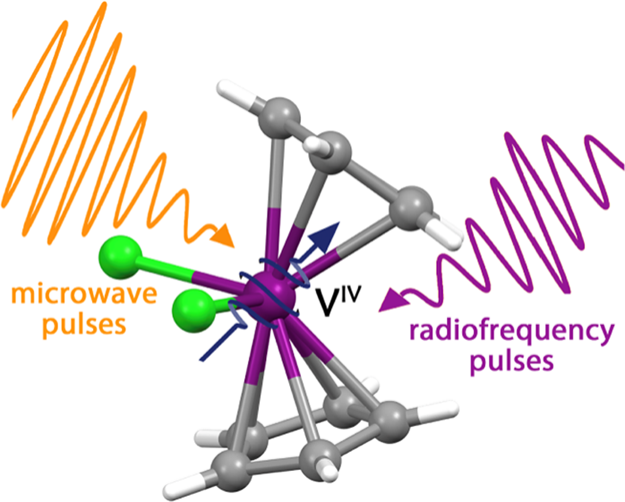

We report here a comprehensive characterization of a 3d organometallic complex, [V(Cp)_2_Cl_2_] (Cp = cyclopentadienyl), which can be considered as a prototypical multilevel nuclear qudit (nuclear spin *I* = 7/2) hyperfine coupled to an electronic qubit (electronic spin *S* = 1/2). By combining complementary magnetic resonant techniques, such as pulsed electron paramagnetic resonance (EPR) and broadband nuclear magnetic resonance (NMR), we extensively characterize its Spin Hamiltonian parameters and its electronic and nuclear spin dynamics. Moreover, we demonstrate the possibility to manipulate the qubit–qudit multilevel structure by resonant microwave and radiofrequency pulses, driving coherent Rabi oscillations between targeted electronuclear states. The obtained results demonstrate that this simple complex is a promising candidate for quantum computing applications.

## Abstract 2

[V(Cp)_2_Cl_2_] (Cp = cyclopentadienyl) can be considered as a prototypical multilevel nuclear qudit (nuclear spin *I* = 7/2) hyperfine coupled to an electronic qubit (electronic spin *S* = 1/2). By combining pulsed EPR and broadband NMR, its Spin Hamiltonian and electronic and nuclear spin dynamics are characterized. The study demonstrates the possibility to manipulate the qubit−qudit multilevel structure by resonant microwave and radiofrequency pulses, driving coherent Rabi oscillations between targeted electronuclear states.

## Introduction

In the past few years, the research on *S* = 1/2 molecular spin systems, which can be used as the building blocks of a potential quantum computer, experienced a surge of interest.^1−3^ Such elementary units are two-level quantum systems called qubits. Both transition metal and organic radical molecule-based qubits with coherence times long enough to allow single-qubit gate manipulation have been recently reported.^4−14^ Molecule-based two-qubit gates were also realized by linking different paramagnetic units (each encoding a qubit) and engineering the coupling between them.^15−17^

A distinctive feature of molecular spin systems, compared to other consolidated platforms for quantum computing (QC),^18−27^ is the ease of obtaining single-quantum objects featuring more than two levels: such systems, with a number of degrees of freedom *d* > 2, are known as qudits.^28−35^ Their multilevel structure can be characterized in detail by exploiting different experimental and theoretical techniques,^36−38^ and states encoded in such levels can be manipulated by electromagnetic pulses in the microwave^39−43^ or radiofrequency ranges.^44^ This capability of encoding and manipulating the state of a qudit places molecular spins among the most promising platforms for the actual realization of a quantum computer. Indeed, the additional levels available within each logical unit could significantly simplify the implementation of several QC codes (in terms of the number of both required units and operations),^28,31,33,45−47^ making it potentially achievable in the short term. An outstanding example is the Grover search algorithm, which was recently implemented using the four levels of a nuclear spin 3/2 as the quantum space search.^48^

Qudits have the further crucial capability of encoding logical units with embedded quantum-error correction (QEC), as recently shown by some of us for molecular systems.^49^ One of the major issues to be solved in the perspective of actual quantum computation is indeed the protection, through QEC, of quantum information from its intrinsic fragility. Such a protection is attained by encoding information in so-called “logical qubits”, which, at variance with simple qubits, feature more than two degrees of freedom. Molecular qudits offer the natural solution to this issue,^29,44,49−52^ without resorting to multiqubit encodings in which many physical units are used to encode a single logical one, an extremely impractical route for near-term QC architectures.^53^

In this respect, molecules are extremely attractive in light of their versatility: the combination of more than one paramagnetic center in molecular architectures with defined and tailored exchange coupling can provide high-spin ground states well separated in energy from excited ones. This can be further split, due to magnetic anisotropy, in a series of sublevels not equally spaced in energy, an essential feature for QEC schemes since it allows selective targeting of different transitions. Furthermore, the molecular nature of these systems makes them highly tunable in terms of the properties and the fulfilling of specific requirements to be met for quantum computation schemes.^15,54−57^

An even simpler realization of a molecular qudit can be obtained considering molecules characterized by an electronic or nuclear spin > 1/2. The former condition (*S* > 1/2) can be easily matched in complexes containing a single magnetic ion (such as Cr^3+^, *S* = 3/2, or Fe^3+^, *S* = 5/2) properly coordinated to the surrounding ligands to achieve a long coherence time.^40,44,58−61^ The latter scenario (*I* > 1/2) is easily obtained for some isotopes and is intrinsically protected from decoherence, thanks to the weak coupling of the nuclear spin to the environment, which also results in long relaxation times. On the other hand, manipulation of nuclear spins is impractically slow due to their weak magnetic moment. Such a drawback can be overcome by using nuclear spin qudits coupled to electronic spins by hyperfine interactions, which speeds up nuclear transitions through electronuclear mixing.^44^ This coupling is also particularly useful in some QEC algorithms (e.g., using the electronic spin as an ancilla for error detection)^49^ or quantum simulation algorithms involving the interaction of an atom (encoded in a spin 1/2) with a radiation field (encoded in the multilevel qudit).^53^

Vanadium(IV)-based complexes are among the most interesting molecules to be investigated along this line of research. Indeed, the natural abundance of the ^51^V isotope, a *I* = 7/2 nuclear spin, which can encode an 8-level qudit, is close to 100% and these complexes can show remarkably long electronic and nuclear phase memory times.^9−12,39,41,62^ Recent studies further pointed out that in spite of the small quadrupole interaction typical of ^51^V nucleus (and 3d metal ions in general), hyperfine coupling yields a pattern of levels which can be reproduced by an effective quadrupolar splitting along specific magnetic field directions.^63^ This energy level structure gives full control of the system by radiofrequency (rf) pulses resonant with single-quantum transitions.^64^

Among the class of complexes which have not yet been considered for this scope a prominent place is occupied by organometallic systems. In this respect, some of us recently reported the first characterization of electronic spin decoherence in an organometallic titanium-based complex, which shows very appealing properties despite its large number of hydrogen atoms.^65^ This was attributed both to geometrical (hydrogen atoms being located inside the spin diffusion barrier) and vibrational factors, with spin–lattice relaxation remaining long enough not to affect the phase memory time due to the peculiarly weak spin–phonon coupling of the low energy vibrations.^65^

These results prompted us to extend the study of the nuclear and electronic decoherence to vanadium-based organometallic systems for which, notwithstanding earlier continuous-wave (CW) EPR characterization, the analysis of spin dynamics has not been reported. Starting from the synthesis, the crystal structure and CW-EPR spectra reported in the seventies’,^66,67^ we performed a thorough experimental study on single crystals and powder of [V(Cp)_2_Cl_2_] (Cp = cyclopentadienyl), hereafter **1**, by CW- and pulsed-EPR and broadband nuclear magnetic resonance (NMR).^68^ This system was chosen in virtue of its uncommon air stability, the possibility of easily obtaining single crystals of relevant size, and the ease of dilution in a diamagnetic isostructural analogue, namely, [Ti(Cp)_2_Cl_2_], hereafter 2. Furthermore, the easy replacement of chloride ligands with a bridging ligand enables in perspective the preparation of multiqudit quantum gates.

In this contribution, we thus characterize in detail its Spin Hamiltonian, both in its electronic and nuclear part, including the weak quadrupolar interactions, and address single-quantum coherences and Hahn echo decay studies as a function of the external magnetic field. This allows the determination of electronic and nuclear coherence times which is comparable to those observed in nonorganometallic complexes. We further demonstrate that at relatively low magnetic fields, the anisotropic hyperfine coupling has the same effect of a sizable nuclear quadrupole coupling, i.e., it results in nuclear spin sublevels not being equally spaced. This energy pattern allows us to individually address by NMR each nuclear spin transition.^63,64^ Finally, we report on the possibility of observing both nuclear and electronic Rabi oscillations, indicating the possibility of creating any arbitrary superposition of states, either nuclear or electronic. These properties make [V(Cp)_2_Cl_2_] a promising candidate to implement qudit-based quantum algorithms and QEC schemes.

## Results and Discussion

### Molecular and Crystal Structure

**Figure 1:**
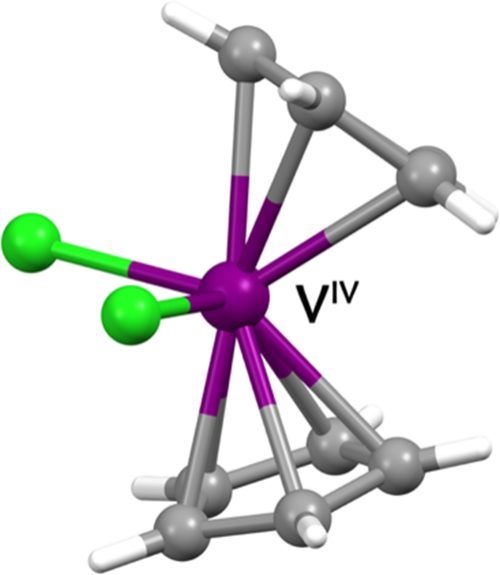
View of the molecular structure of **1**. Color codes: purple, vanadium; light green, chlorine; gray, carbon; and white, hydrogen.

Previous reports, of which we summarize hereafter the main findings, indicated that **1** crystallizes in the monoclinic space group n° 14, in which each unit cell contains two crystallographically independent but nearly identical molecules.^69,70^ The molecular structure of **1** is that of a bent-metallocene complex with the central metal ion coordinated by two cyclopentadienyl ligands (Cp) and two additional coordinating ligands, here chloride. The coordination geometry of the metal center is tetrahedral-like, with an overall *C*_*2v*_ idealized molecular symmetry (Figure 1). Each ring is bent at ca. 24° with respect to the plane passing through the two chlorine and the vanadium(IV) atoms (Figure 1). Consequently, the Cp–V–Cp angle is ca. 132°. The V–Cl distances were reported to be ca. 2.36 Å, and the V–Cp distances ca. 2.05(1) Å for both independent molecules.

The corresponding diamagnetic titanium(IV) derivative, **2**, which was used to obtain diluted solid solutions of **1** (vide infra), crystallizes in the triclinic **P**1̅ space group, with two independent molecules in the unit cell. The titanium(IV) and vanadium(IV) compounds are virtually isostructural (Figure S1).^71^ We report the salient feature of their packing in Figure S2, which shows a view of this crystal structure along the *b* crystallographic axis, evidencing the relative orientation of crystallographically independent molecules in the unit cell. The molecules are packed in layers that grow along the (101̅) crystallographic plane with C···C distances (ca. 3.24 Å) shorter than the van der Waals radii between Cp rings oriented in a face-to-face manner (Figure S2).

Three different crystalline dispersions of **1** in **2** with nominal concentration 10% (**3a**), 1% (**3b**), and 0.1% (**3c**) were prepared to obtain a well-defined set of Spin Hamiltonian parameters through CW-EPR spectroscopy and to investigate the role of different concentrations on the electronic relaxation times. The experimental details for their preparation are reported in the Experimental Section. The crystalline phase of each dispersion was assessed by powder X-ray diffraction analysis (Figure S3), and the nominal concentration of the paramagnetic component **1** was determined through inductively coupled plasma (ICP) atomic emission spectroscopy. This provided effective concentrations of 13.9, 1.13, and 0.10%, for **3a**, **3b**, and **3c**, respectively.

Slow crystallization of the dispersions **3a**–**3c** produces large rectangular prismatic single crystals that preferentially grow along the (111) crystallographic plane. An optical image of a typical crystal of **3c** and faces indexing as found from single-crystal X-ray diffraction is reported in Figure S4.

### Determination of Spin Hamiltonian Parameters

**Figure 2:**
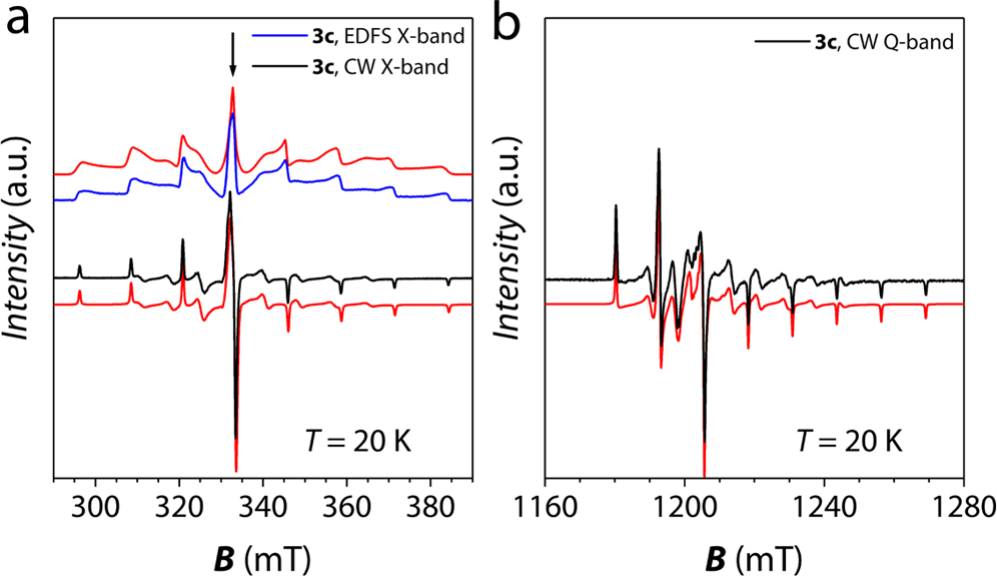
Experimental and simulated EPR spectra for **3c** at *T* = 20 K. The spectral simulations corresponding to the Spin Hamiltonian parameters reported in the text are shown as red lines. (a) X-band Echo detected field swept (EDFS, blue line; ν = 9.70 GHz) and CW (black line, ν = 9.405 GHz) X-band spectra: EDFS data were translated for ease of comparison with CW data (b) CW- Q-band spectrum (black line, ν = 33.7 GHz). The arrow indicates the transition probed for pulsed EPR experiments (see below).

CW-EPR X-band (9.405 GHz) and Q-band (33.7 GHz) spectra of microcrystalline powder dispersions **3a**–**3c** were collected at room temperature. Figure S5 shows the corresponding X-band spectra of the most concentrated dispersions (**3a** and **3b**), while the X and Q-band spectra of the most diluted one (**3c**) are reported in Figure 2.

As previously reported in the literature for a frozen solution of **1**,^66^ compound **3c** shows a CW-EPR spectrum with the expected eight-fold hyperfine splitting typical of V^IV^ (^51^V *I* = 7/2, natural abundance 99.76%) and rhombic **g** and **A** tensors. Peaks due to the largest component of the hyperfine coupling are observed at low and high field values, whereas those due to the smallest component are unresolved in the central region of the spectrum (Figure 2).

For the simulation of EPR spectra at X- and Q-band, we retained only the first two terms (electron Zeeman and hyperfine coupling) of the complete Spin Hamiltonian (eq 1), where **g** is the electronic anisotropic Landé tensor, **A** is the hyperfine coupling tensor, **P** is the quadrupolar interaction, and *g*_*N*_ is the nuclear spectroscopic factor.
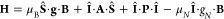
1The spectra of **3c** can be satisfactorily simulated^72^ at both frequencies assuming a collinear rhombic model (i.e., *x* ≠ *y* ≠ *z*) with the following parameters: *g*_*x*_ = 2.0010(5), *g*_*y*_ = 1.9834(5), and *g*_*z*_ = 1.9721(5) and |*A*_*x*_| = 60(2) MHz, |*A*_*y*_| = 216(2) MHz, and |*A*_*z*_| = 348(2) MHz. This set of parameters allows the proper simulation of the overall experimental spectrum without assuming any difference between the two inequivalent molecules in the unit cell. This behavior is not surprising given their very similar coordination environment (vide supra) and because they only differ in their orientation within the crystal, which, however, does not affect the spectra of microcrystalline powder samples. An upper bound on the super-hyperfine coupling constant between the electronic spin and the Cl nuclei (^35^Cl and ^37^Cl, natural abundance 76 and 24%, respectively, *I* = 3/2) was estimated on the basis of the observed linewidths, providing values of the order of 12.6, 6.0, and 0.1 MHz along *x*, *y*, and *z*, respectively. These findings are in general agreement with what was reported in the literature for the same system and closely related compounds.^66,67,73^

**Figure 3:**
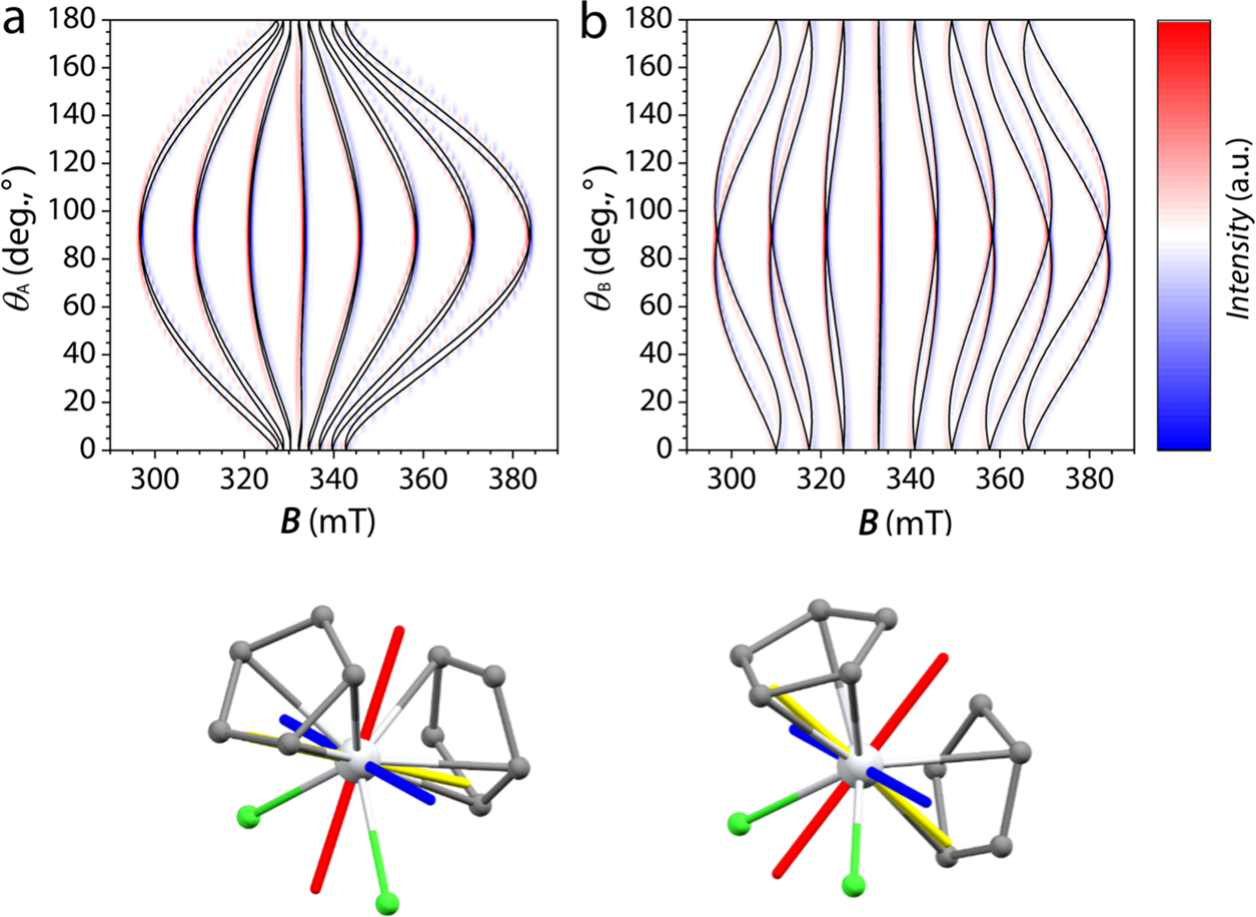
Upper panel: Angular dependences of the single-crystal EPR spectra (X-band) for **3c** at room temperature for rotation A (a) and rotation B (b) (see the text). Red and blue indicate positive and negative values of the EPR signals, respectively, while the angular dependence simulations for the two independent [V(Cp)_2_Cl_2_] molecules are reported in black. Lower panel: orientation of the principal directions of **g** and **A** tensors with respect to the two crystallographically independent molecules of **3c**. Color codes: pale gray spheres, vanadium; green, chlorine; gray, carbon; blue rods: *x* components; red rods: *y* components; and yellow rods: *z* components.

The well-defined shape of the crystals of **3** offers the possibility to proceed with single-crystal EPR studies to characterize the two crystallographically inequivalent molecules. The crystal morphology of **3** (vide supra) provides the most obvious crystal rotations around the edges between the (010) (or (01̅0)) and (111) faces (rotation A) and between the (1̅01) (or (101̅)) and (111) faces (rotation B), respectively (Figure S4). For both rotations, we started with the crystal face corresponding to the (111) crystallographic plane parallel to the magnetic field **B** (θ = 0°) up to θ = 180°. This defines an *XYZ* orthogonal reference frame, with rotation A being performed from *X* to *Z* and rotation B from *Y* to *Z*. The angular dependencies for the two rotations are reported in Figure 3, while the selected single-crystal spectra are reported as an example in Figures S6 and S7.

Both rotations show at θ = 0 and 90° a single set of an eight-fold EPR signal resulting from the hyperfine interaction between the *S* = 1/2 electronic spin of vanadium(IV) and its *I* = 7/2 nuclear spin (vide supra). This observation confirms that the Spin Hamiltonian parameters of the two molecules are indistinguishable and, at these orientations, the magnetic tensors of the two molecules are equally oriented with respect to the magnetic field. In other words, despite the two molecules being crystallographically independent, they are equivalent within the EPR linewidth when measured along these directions. This led us to assume, for the sake of simplicity, that the tensors on the two molecules are related by a binary axis despite the triclinic symmetry. Furthermore, the effective hyperfine splitting decrease in the order *Z* > *Y* > *X*, with the spectrum observed along *Z* being close to the maximum extension (Figure S7). It is also evident from the angular dependencies that while throughout rotation A the two signals are barely distinguishable, throughout rotation B, they are well separated, except at 0 and 90° (Figure S6). This is at variance with what was previously reported in the literature^67^ but consistent with a later paper on the closely related compound [V(CpMe)_2_Cl_2_].^73^ Finally, we note that the observed extreme resonant field values in the two rotations coincide with the position of the lines in the powder spectrum, thus providing a fundamental clue to determine the orientation of the principal axes of the magnetic tensor. Accordingly, the angular dependencies of the resonant fields were well reproduced on the basis of the Spin Hamiltonian parameters obtained by the powder study and using magnetic tensors orientation similar to that derived for [V(CpMe)_2_Cl_2_]: within the experimental error (2°) associated with our experimental setup for one of the two possible molecule/tensor assignments *z* components of the tensors are perpendicular to the Cl–V–Cl plane, *y* ones lies along the bisector of the Cl–V–Cl angle, and *x* are along the direction orthogonal to these two (Figure 3 and Table 1).

**Table 1:** Principal Values of the Magnetic Tensors of **3a–3c** as Determined by the Combined EPR and NMR Analyses

	tensor		
component	**g**	**A** (MHz)	**P** (MHz)
*x*	2.0010(5)	–55(2)	0.09(1)
*y*	1.9834(5)	–216(2)	0.09(1)
*z*	1.9721(5)	–351(2)	–0.18(1)

The single-crystal EPR study thus provided a sound set of electronic spin Hamiltonian parameters and solved the issue related to the orientation of the anisotropy axes in this molecule.^67,73^ Indeed, refs (67) and (73) reported remarkable differences in the orientation of the anisotropy axes with respect to the molecular structure despite the similarity of the investigated complexes. This has to be attributed to the absence of a reliable structure determination for **1** and **2** at the time of ref (67). To directly investigate the nuclear Spin Hamiltonian parameters and the orientation of the magnetic tensors, single-crystal ^51^V (*I* = 7/2, γ/2π = 11.21 MHz/T) NMR spectra were subsequently recorded at a fixed temperature of *T* = 4 K, in the **B** = 0–0.4 T range on single crystals of **3b**. The magnetic field was applied along three independent directions, corresponding to the normals to the indexed crystal faces, as depicted in Figure S8. For the sake of simplicity, we will refer to these directions as **B**_S_, **B**_M_, and **B**_L_, for the magnetic field applied along the shortest, medium, and longest crystal dimensions, respectively.

**Figure 4:**
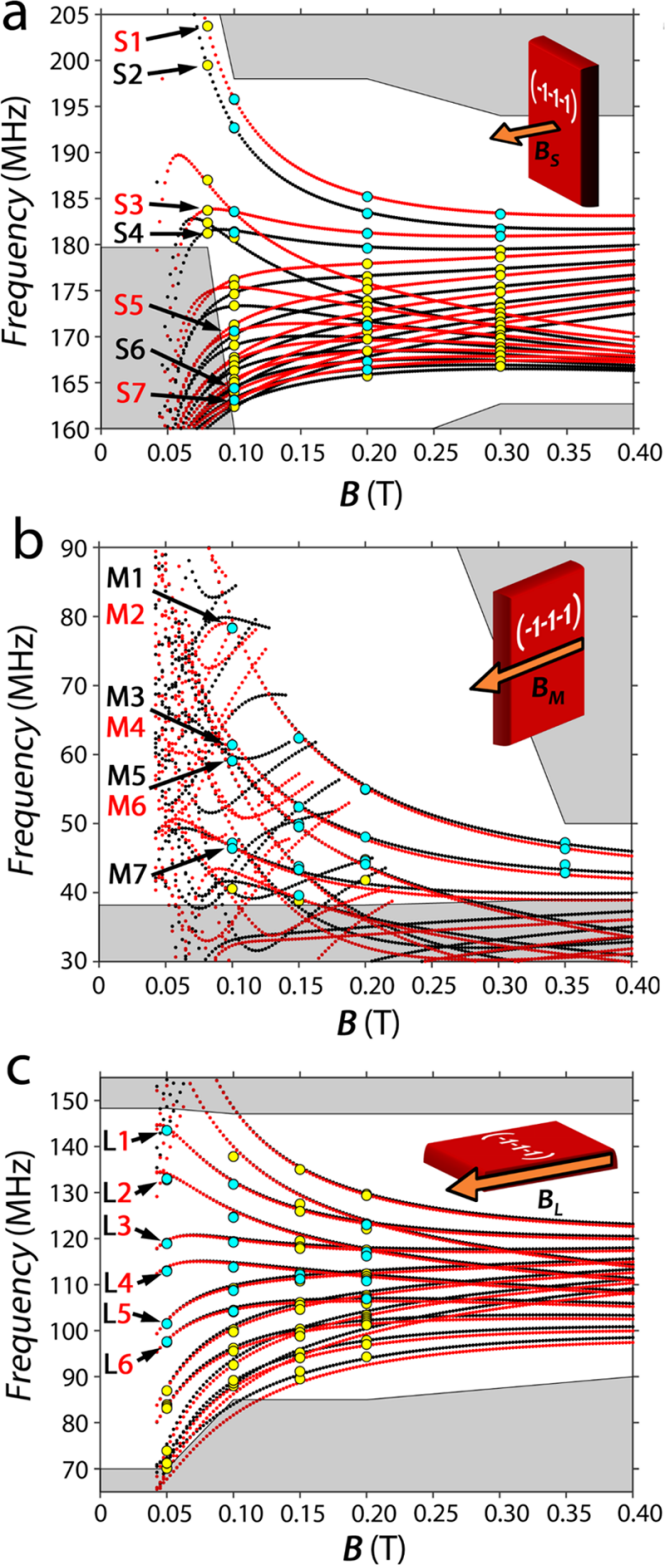
Measured (yellow and cyan dots) and calculated (red and black dots) NMR frequencies of the two inequivalent molecules in **3b** for three different orientations of the static magnetic field with respect to the single crystal at *T* = 4 K (examples of spectra as a function of frequency are reported in Figure S9). Experimental data highlighted in cyan represent the spectral peaks for which the nuclear phase memory time ^*n*^*T*_2_ has been measured (labeled transitions). The S, M, and L labeling of the transitions refers to the applied magnetic field directions. Red and black dots refer to the two inequivalent molecules. Shaded gray areas were not experimentally accessible or have not been explored.

The positions of the peaks in the NMR spectra have been used, together with the best-fit parameters of the EPR investigation, to refine the values of the parameters of the complete Spin Hamiltonian (eq 1). For the simulation of the NMR data, we assumed the same set of principal values for the two inequivalent molecules as derived from the EPR spectra. To account for small experimental errors on crystal orientation, we let the magnetic field orientation free to refine around the nominal directions (Table S1), while we fixed the principal direction of the tensors with *y* components exactly along the bisector of Cl–V–Cl and the *z* one perpendicular to the same plane, for each of the two molecules: i.e., we dropped the pseudo-monoclinic point symmetry assumed in the analysis of EPR data. The resulting tensor orientations with respect to the *ab*′c* orthogonal system compare very well with those determined by EPR (Table S2), and the refined orientation of the crystal indicates that the misalignment with respect to the idealized directions reported in the inset of Figure 4 is minimal (less than 1°). As for the principal values of the tensors, those of **g** were kept fixed with respect to the values obtained by EPR spectroscopy, while those of **A** were varied to match the frequency evolution with the static magnetic field of the modeled low-temperature transitions.

The full set of observed NMR peaks as a function of the static magnetic field **B** is reported in Figure 4 for the three different orientations (**B**_S_, **B**_M_, and **B**_L_) with respect to the crystal frame; the experimental data are optimally reproduced using the following set of parameters: *g*_*x*_ = 2.0010(5), *g*_*y*_ = 1.9834(5), and *g*_*z*_ = 1.9721(5); *A*_*x*_ = −55(2) MHz, *A*_*y*_ = −216(2) MHz, and *A*_*z*_ = −351(2) MHz; and *p*_*x*_ = 0.09(1) MHz, *p*_*y*_ = 0.09(1) MHz, and *p*_*z*_ = −0.18(1) MHz. The difference between the |*A*_*z*_| values determined by EPR and NMR is negligible (lower than 1%), whereas NMR allowed measuring the *A*_*x*_ component with higher accuracy given its higher resolution. Moreover, NMR allows determining the absolute sign of all of the hyperfine tensor components, which are not accessible by CW-EPR. We notice that the trace of the hyperfine tensor agrees, within experimental accuracy, with literature EPR data on diluted glasses of this molecular complex.^66^ The set of Spin Hamiltonian parameters is also consistent with expectations based on simple electronic structure theory and indicates a ground state which is mainly d_*z*^2^_ with relevant contribution from d_*x*^2^–*y*^2^_.^66^ Finally, we remark that an axial quadrupolar tensor **P** proved to be essential to reproduce the NMR spectra. This term, together with the second-order contribution of the transverse hyperfine coupling (see ref (64)), is responsible for the differences between the subsequent nuclear gaps and consequently for the spectral resolution of nearest transitions. This ensures the ability to finely address and manipulate single nuclear transitions (vide infra).

**Figure 5:**
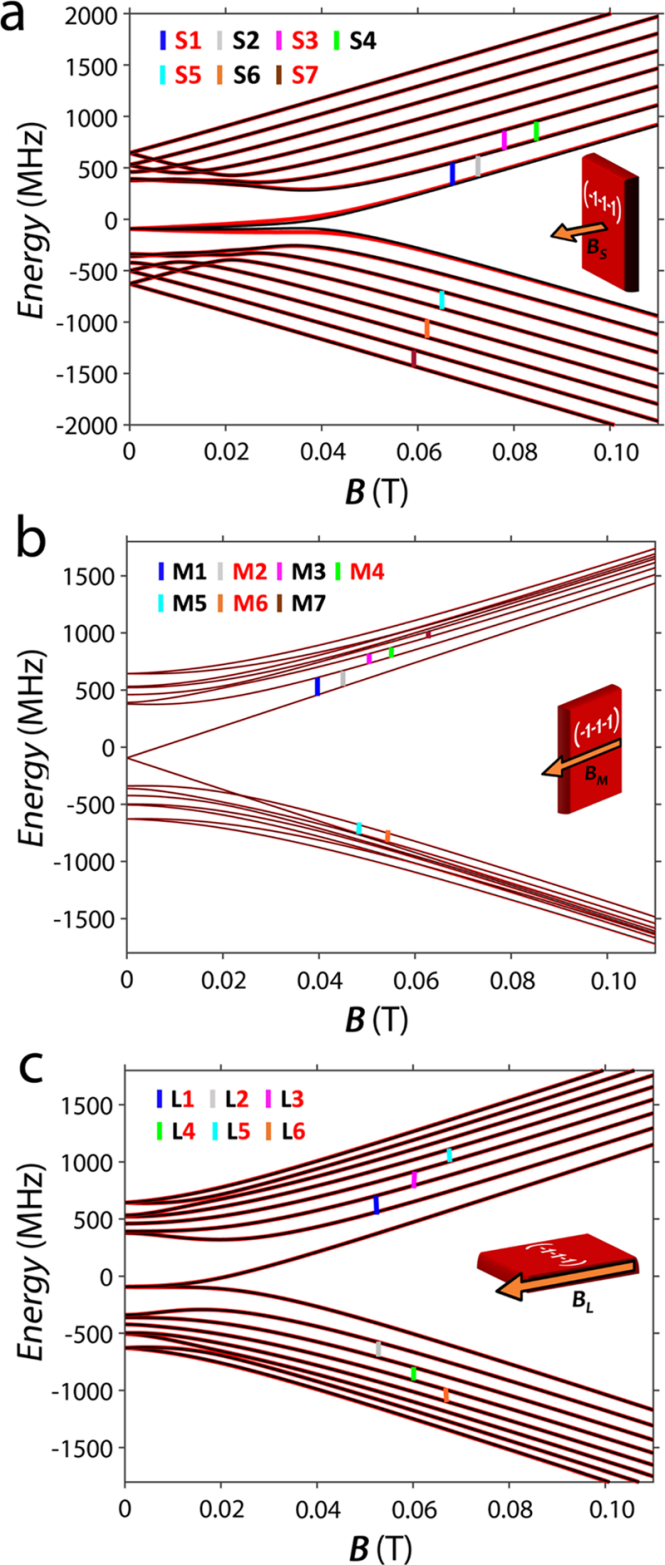
Energy level diagrams of **3b** as a function of the field for the three directions of the static magnetic field, as described above (**B**_S_, **B**_M_, and **B**_L_). Red and black lines represent the calculated eigenvalues for the two inequivalent molecules in the unit cell, while the vertical-colored bars indicate the transitions for which the nuclear relaxation rates ^*n*^*T*_2_ were measured (see Figures 7 and S14).

The energy level diagrams as a function of the field obtained by diagonalization of the full Spin Hamiltonian (eq 1) are reported in Figure 5 for the three orientations **B**_S_, **B**_M_, and **B**_L_. They show a similar behavior for the two molecules in the unit cell along all of the explored directions of the static magnetic field. However, the slightly different energy gaps result in distinct transition manifolds, mostly distinguishable with the NMR experiment frequency resolution.

The difference between the transition energies of the two inequivalent molecules in the unit cell depends on the directions and intensity of the static magnetic field. For instance, when the magnetic field is oriented along **B**_L_, the spectra are almost identical in the low magnetic field regime, while when the magnetic field is oriented along **B**_S_, differences of up to 5 MHz are observed. This is consistent with the small deviation with respect to binary symmetry of the relative orientation of the two molecules and thus of their respective magnetic tensors, which cannot be appreciated by EPR.

### Electronic and Nuclear Spin Dynamics

To investigate the temperature dependence of the electronic relaxation times of the crystalline dispersions **3a–3c**, X-band Echo detected field swept (EDFS) EPR spectra were recorded on powder microcrystalline samples using a standard Hahn echo sequence. Figure 2 reports, as an example, the data obtained at 20 K. As evidenced by the presence of an intense spin-echo, we can anticipate that quantum coherence is expected for all of the measured samples. Moreover, the spin Hamiltonian parameters obtained by the analysis of the CW-EPR spectrum provide a good simulation of the EDFS spectra, indicating that all of the allowed electron spin transitions of **3** are detected under the experimental conditions (Figure 2).

**Figure 6:**
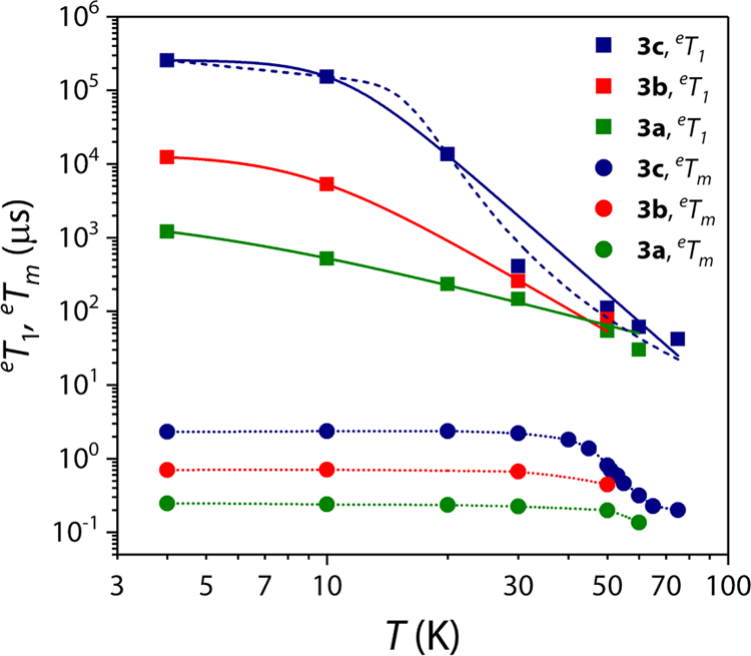
Temperature dependence of ^e^*T*_1_ and ^e^*T*_m_ for **3a–3c** (see legend). Solid and dashed lines are the best fit of the models using eqs 3 and 4, respectively. Dotted lines are a guide for the eye. Error bars are within the size of the symbols.

Inversion recovery experiments were performed in the 4.5–75 K temperature range for **3a**, **3b**, and **3c** at the X-band frequency to investigate the temperature dependence of the electronic spin–lattice relaxation time ^e^*T*_1_. To obtain a signal as large as possible and for the sake of comparison with the data reported on vanadyl and vanadium(IV) tris-chelate complexes, the experiments were conducted in an external static field of 0.334 T, corresponding to the most intense transition of the powder spectrum. This corresponds to the *m*_I_ = −1/2 transition, where all molecules are excited due to the negligible angular dependency of the resonance field (see Figure S10). The resulting inversion recovery traces (Figure S11) were fitted with a stretched monoexponential equation
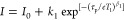
2and the extracted ^e^*T*_1_ values are reported in Figure 6.

As expected, maximum ^e^*T*_1_ values are observed at the lowest temperature (4 K) for the most diluted crystalline dispersion (**3c**), with ^e^*T*_1_ that decreases by ca. 1 order of magnitude passing from **3c** to **3b** and from **3b** to **3a**. On increasing the temperature, ^e^*T*_1_ values remain almost constant up to 10 K, and then they decrease down to ca. 100 μs at 75 K, with small differences as a function of the concentration of the crystalline dispersion. This temperature dependence, which is clearly observed for **3c** and to a lesser extent for **3b** and **3a**, is due to the faster relaxation induced by the higher paramagnetic concentration typical for vanadium(IV) complexes. At first, the temperature dependencies of ^e^*T*_1_ were modeled by assuming two contributions to the relaxation: a direct one, dominating at low temperatures, and a Raman-like one dominating at high temperatures
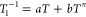
3A reasonable but not entirely satisfactory fit of the data (solid lines in Figure 6) was achieved with the values of the Raman-like exponent *n* of 4.8, 3.2, and 2.44 for **3c**, **3b**, and **3a**, respectively (Table S3). Even if the limited number of experimental points and the only partial agreement with the highest temperature data suggest that the values of these parameters might not be completely meaningful, the value of *n* ca. 5 for the more detailed set of data, **3c**, is consistent with the observed rapid decrease of ^e^*T*_1_ with temperature. This value is to be compared with the typical values of *n* around 3 for square-pyramidal vanadyl complexes and of *n* of ca. 4 for octahedral tris-chelated vanadium(IV) complexes. The less abrupt temperature dependence of ^e^*T*_1_ for the former results in measurable ^e^*T*_m_ up to room temperature, whereas for the latter, the collapse of ^e^*T*_1_ results in ^e^*T*_m_ being unmeasurable above 150 K.^10^ In this respect, the fast decrease of ^e^*T*_1_ observed in **3c** is detrimental for its perspective use as a spin qubit at high temperatures. The concentration dependence of ^e^*T*_1_ deserves some comment. The best-fit parameters reported in Table S3 show that varying the level of doping affects both Raman and direct processes. At low temperatures (*T* < 20 K), it is obviously the direct term that dominates, whereas at higher temperatures, the Raman prevails. We tentatively attribute this behavior of spin–lattice relaxation to the difference in dipolar interactions. It is indeed well-known that these interactions can provide efficient relaxation pathways in the solid state.

A more detailed model involving a direct mechanism of relaxation at low temperatures and a local vibrational mode responsible for the high-temperature relaxation^74,75^ has also been used (eq 4)
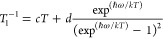
4This model satisfactorily reproduces the temperature dependence of *T*_1_ for **3c**, especially the drop in *T*_1_ observed between 30 and 40 K. It also provides the frequency (ℏω = 120 cm^–1^) of a potential low-frequency phonon involved in the relaxation. While phonons responsible for the relaxation in these compounds are typically in the 20–50 cm^–1^ range, it is not unusual to observe more than one phonon involved in the relaxation, the second one in the 100–200 cm^–1^ energy range.^62^ Since a model involving two local-modes (ℏω_1_ and ℏω_2_) cannot be used here for the limited number of experimental points available, and given the absence of any experimental or theoretical determination of vibrational frequencies for this molecule, we can only speculate that the best-fit value might actually result as an average value of the energies of the two lowest-lying modes.

To investigate the quantum coherence in detail and to quantify the electronic phase memory time of **1** in the powder microcrystalline dispersions **3a**, **3b**, and **3c** as a function of the temperature, echo decay experiments were also performed at the same field of the inversion recovery experiments. The decay traces (Figure S12) were fitted using the stretched-exponential equation
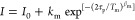
5as usually done for transition metal systems, where *I* indicates the echo intensity, 2τ_p_ is the delay between the initial pulse and the echo detection, and β_m_ is the stretch factor. The temperature dependence of ^e^*T*_m_ for **3a–3c** is reported in Figure 6.

The temperature dependences of *T*_m_ for all crystalline dispersions show a temperature-independent behavior in the 4.5–40 K range, with values of ^e^*T*_m_ of 2.3, 0.70, and 0.25 μs, for **3c**, **3b**, and **3a**, respectively. Then, they abruptly decrease as the temperature increases, reaching values of ca. 0.1–0.2 μs at 75 K for all crystalline dispersions (Figure 6). The low-temperature values of ^e^*T*_m_, especially that of the most magnetically diluted crystalline dispersion **3c**, compare well to typical values observed for vanadium(IV)-based potential molecular qubits,^10,11^ with a remarkable exception of the nuclear spin-free system reported by Freedman and co-workers.^9^ This behavior can be surprising at first sight if one considers that **1** features two Cp ligands with a H-rich molecular structure and the spin active nuclei of the two chlorine coligands. Furthermore, while the H atoms of the Cp directly bound to vanadium(IV) are well within the diffusion barrier, those on neighboring molecules should induce a measurable effect. However, the observation of a relevant concentration dependence of ^e^*T*_m_ even below 1% concentration of paramagnetic centers suggests that in this regime, the decoherence is still mostly driven by electronic spin–spin interaction. This is masking the dephasing effect due to electron–nuclear interactions, which are, however, clearly less effective in promoting relaxation. This is at variance with recent observation in titanium(III) organometallic complexes, for which the proton atoms on the neighboring molecules turned out to be the driving force promoting decoherence in the solid solution.^65^ A detailed investigation of such effects would, however, require going to higher dilutions, which is beyond the scope of this article.

An important point to underline is the abrupt drop of ^e^*T*_m_ at ca. 40 K. The drop occurs in a temperature range where ^e^*T*_m_ is not limited by ^e^*T*_1_ but where a marked decrease of the absolute values of ^e^*T*_1_ is also observed (vide supra). This behavior suggests that the two phenomena might be correlated, possibly because a faster ^e^*T*_1_ causes faster electronic fluctuations, which directly impacting ^e^*T*_m_: in other terms, in the expression 1/^e^*T*_m_ = 1/^e^*T*_2_ + 1/2^e^*T*_1_, the spin–lattice term is not negligible at these temperatures despite not being dominant.

To probe the local environment of the vanadium(IV) ion and try to correlate the rapid decrease of ^e^*T*_1_ with (rotational) vibrations, Q-band Mims ENDOR spectra (Figure S13) were recorded at 20 and 50 K, respectively, that is, in the temperature region where ^e^*T*_1_ is almost maximum (20 K) and where its value is reduced by 3 orders of magnitude (50 K).

The ^1^H ENDOR spectra taken at the two temperatures display the same pattern, with a maximum ^1^H coupling of ≈6.2 MHz, with no evidence of motional averaging. Based on a point-dipole approximation and assuming a pure dipolar interaction, these couplings correspond to the shortest V···H distances of the order of 4 Å. This value is consistent with the structural findings, which reveal the shortest V···H distances of 4.3 Å. This seems to exclude that the rapid decrease of ^e^*T*_m_ at relatively low temperatures in this compound is activated by the onset of a temperature-activated rotational vibration.

Overall, even if limited by the temperature dependence of ^e^*T*_1_, the values of electronic phase memory times ^e^*T*_m_ observed at low temperatures for **1** highlight that organometallic vanadium(IV) complexes represent a potential playground for the investigation of quantum coherence in molecular systems.

**Figure 7:**
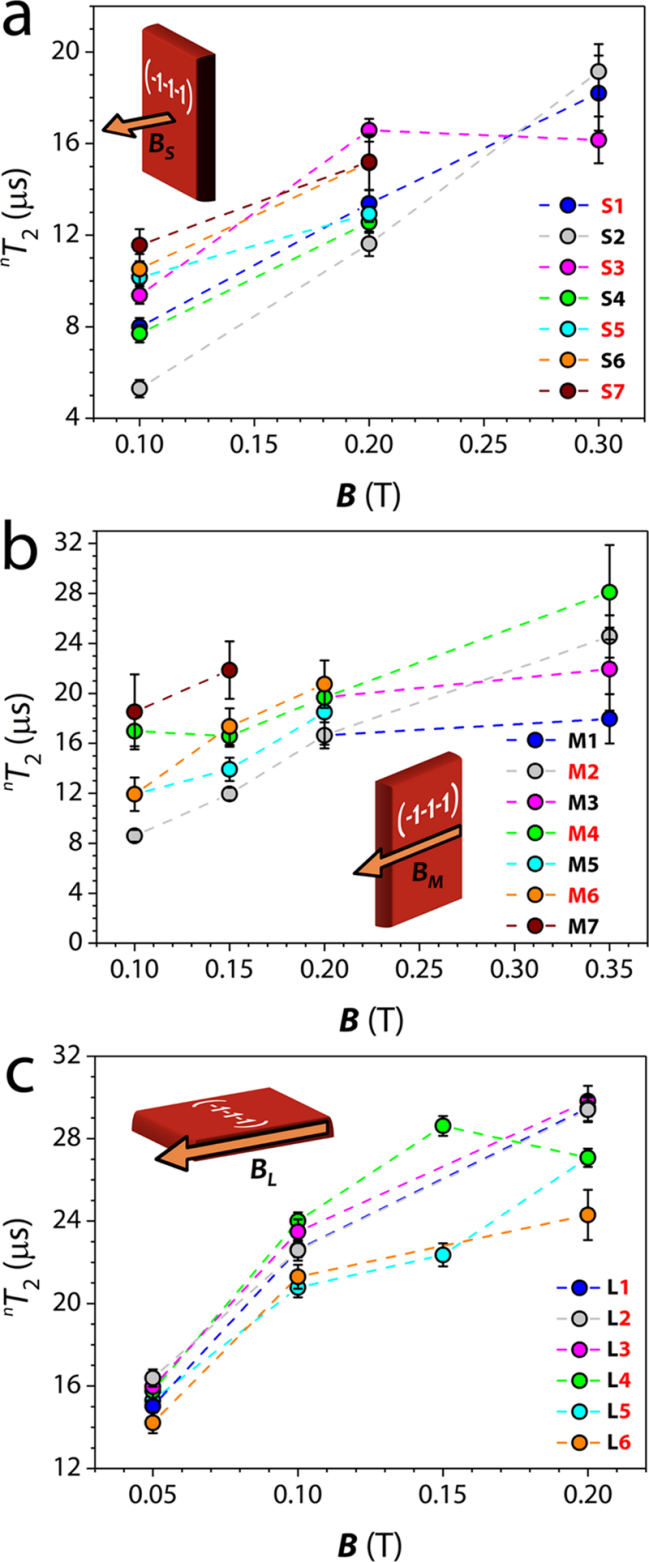
Nuclear spin coherence times ^*n*^*T*_2_ measured at *T*= 4 K with a Hahn echo sequence for the transitions indicated in Figures 4 and 5 at different magnetic field orientations with respect to single crystals of **3b**.

Nuclear phase memory times ^*n*^*T*_2_ were measured for each applied magnetic field for the main spectral lines by recording, as a function of the delay τ, the excited echo intensity on a single crystal of **3b**. The decay of the echo amplitude was fitted with reasonable accuracy by a single exponential law (Figure S14), depicting the evolution of the transverse magnetization (eq 6).
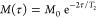
6The resulting field dependence of nuclear coherence times ^*n*^*T*_2_ for all of the identified transitions is reported in Figure 7 for the three static magnetic field directions.

We note that for all of the nuclear transitions analyzed, the observed ^*n*^*T*_2_ increases as a function of the intensity of the static magnetic field. This indicates that the ^51^V nuclear decoherence can be attributed to the interaction with the neighboring nuclei and the dipolar couplings with other electronic spins. The latter is largely reduced in this sample as a consequence of the magnetic dilution (ca. 1%) in the titanium(IV) diamagnetic analogue. The observed increase in ^*n*^*T*_2_ with the magnetic field is consistent with the corresponding increase in electronic polarization and reduction of the electron–nuclear mixing, which reduces nuclear spin-flops. The measured ^*n*^*T*_2_ of ca. 20–30 μs are very competitive if compared to the values obtained for other coupled qubit–qudit systems,^64^ thus providing a sound starting point for system manipulation without significant coherence losses. Longer nuclear decay times with respect to ^e^*T*_m_ (more than 1 order of magnitude) are due to the different origins of the two decoherence processes. Indeed, ^*n*^*T*_2_ is determined by an indirect and less efficient process that involves an effective coupling to neighbor nuclei by means of the interaction with electrons.

### Electronic and Nuclear Coherence

**Figure 8:**
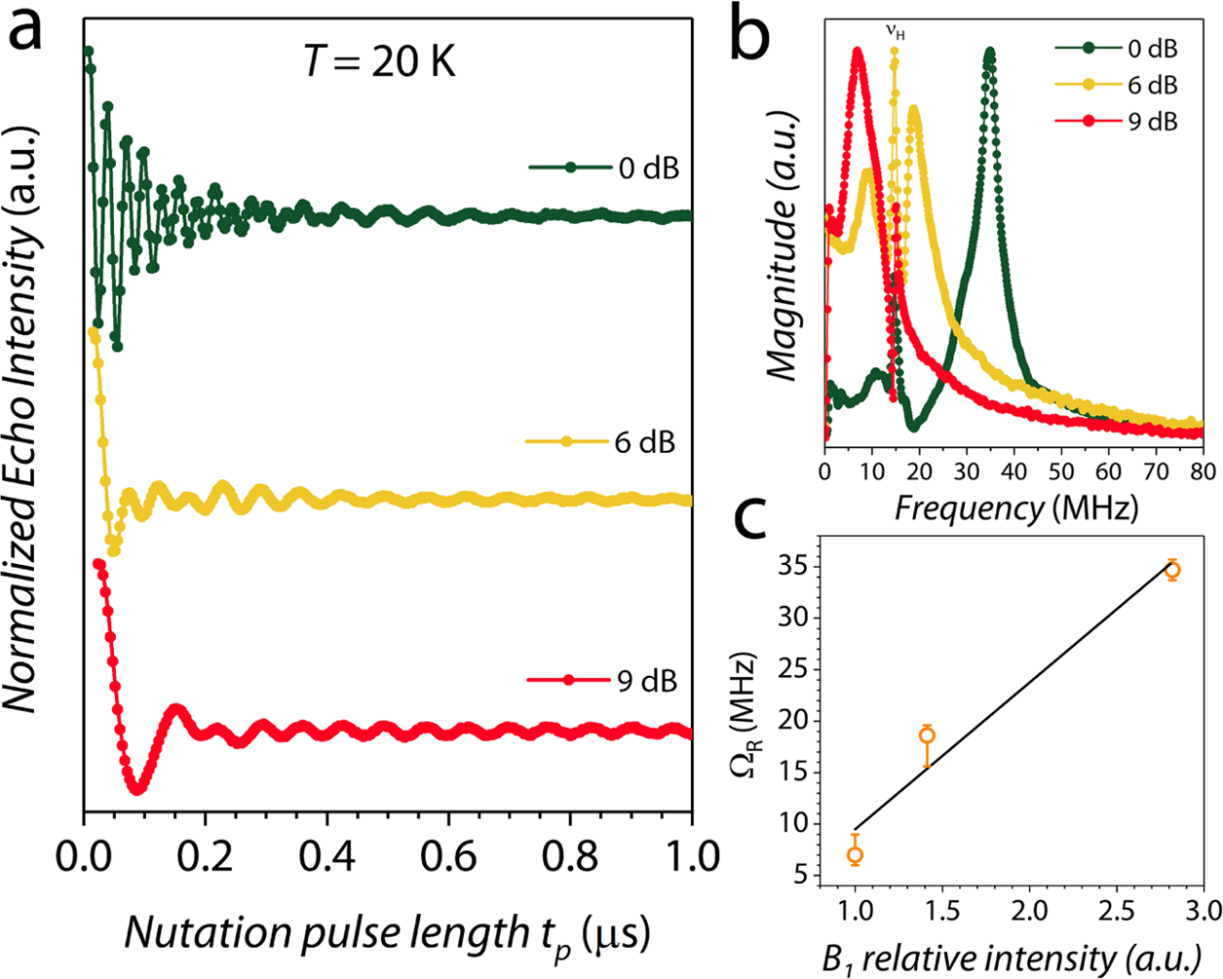
(a) Rabi oscillations recorded for **3c** at *T* = 20 K for different microwave attenuations (X-band). (b) Fourier transform of the Rabi oscillations. (c) Dependence of the Rabi frequency (Ω_R_) on the relative amplitude of the *B*_1_ microwave field and best linear fit (black line). The larger error of Ω_R_ at 6 dB is due to the partial overlap with the proton Larmor frequency (ν_H_), see panel b.

To prove that the observed coherence times allow performing coherent electronic spin manipulations, i.e., to place the spins in any arbitrary superposition of states, nutation experiments were performed at different microwave powers at X-band for the less concentrated crystalline dispersion **3c**. Rabi oscillations were clearly observed at low temperatures with the expected linear dependence of the Rabi frequency, Ω_R_, as a function of the microwave attenuation (Figure 8). All of the frequency domain spectra display a contribution due to the genuine Rabi frequency, which is linearly dependent on the amplitude of the applied microwave field (Ω_R_ = |γ_e_|*B*_1_, see Figure 8c), along with a narrow feature at 14.7 MHz due to the proton nuclear Larmor frequency (ω_N_ = γ_N_*B*_0_ = 2π × 14.7 MHz). This feature is enhanced at 6 dB when the Hartmann–Hahn condition^76^ is fulfilled (Ω_R_ ≈ ω_N_) and indicates a strong coherent coupling between the vanadium electron spin and the proton bath.^77^

**Figure 9:**
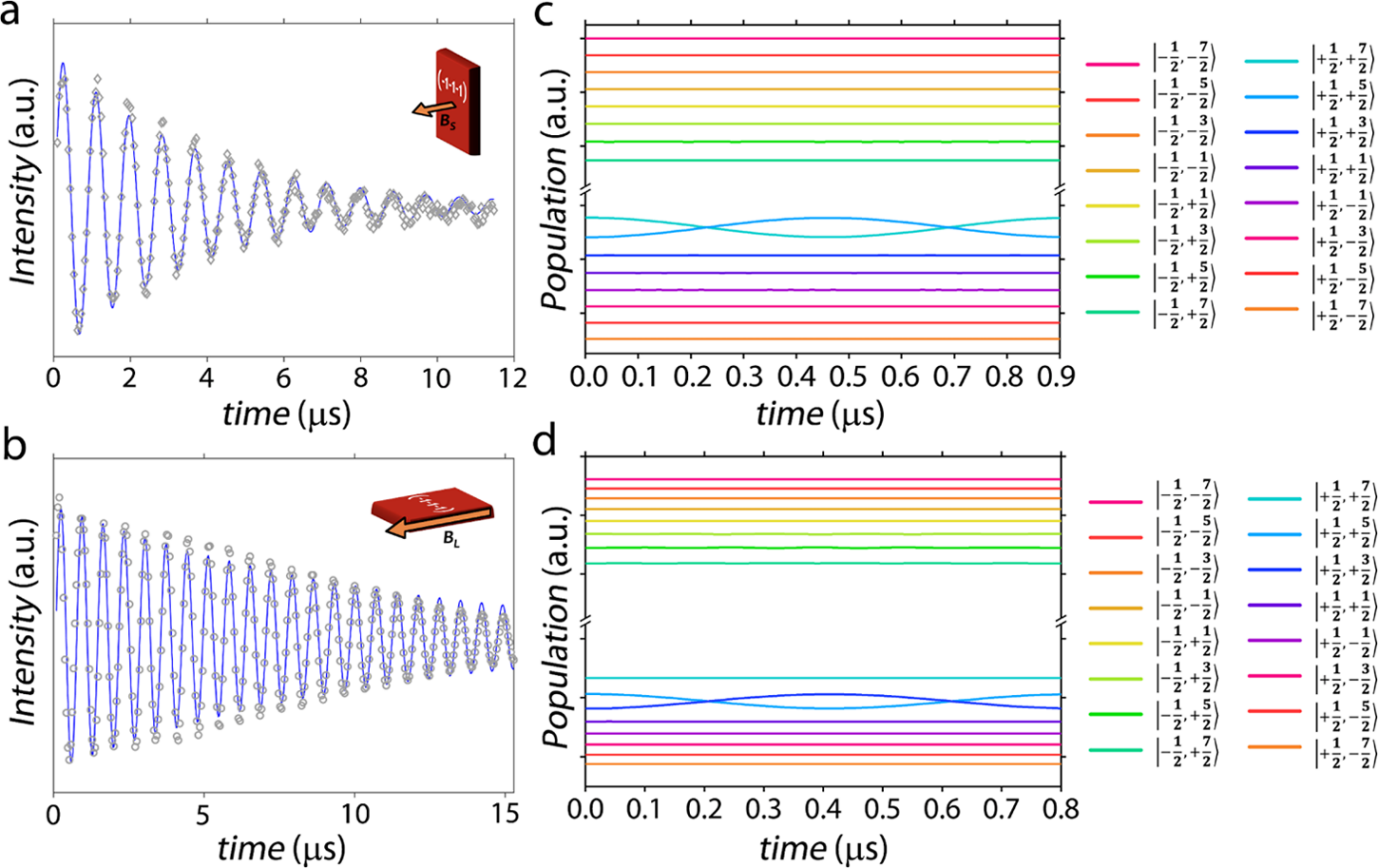
Nuclear Rabi oscillation induced by rf pulse sequences (see the text and Experimental Section) on transitions **S1** (empty circles, panel a) and **L1** (empty circles, panel b) at fixed static magnetic field values (respectively **B**_s_ = **B**_L_= 0.1 T) and *T* = 4 K on a crystal of **3b**. The damping differences between these two measurements, despite similar ^*n*^*T*_2_ values, is attributable to the effect of pulse inhomogeneity along crystal directions with a sizeable difference in length (L ≫ S). Panels c and d show the simulated Rabi oscillations driven on the same experimentally probed transitions **S1** and **L1** in the same experimental conditions in terms of variation in nuclear level populations. The m_I_ labels are correct to 0.5–1% in the external dc field considered.

The possibility to coherently manipulate the qubit–qudit system **1** not only with microwave pulses (mw) (vide supra) but also with radiofrequency (rf) pulses was investigated by NMR. Using this technique we were able to induce Rabi oscillations for various magnetic field configurations. For each static magnetic field direction, Rabi oscillations were induced by a refocusing echo sequence in which the first pulse of increasing length (θ(*t*)) is followed by a refocusing π pulse for echo intensity detection (Figures 9 and S15).

The stimulated echoes were fitted by eq 7, consisting of an oscillating term (Rabi frequency, ^*n*^Ω_R_) and a damping factor λ, depending on both the nuclear phase memory times *T*_2_ and the inhomogeneities of the rf field
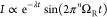
7The enhancement of ^*n*^Ω_R_ with the oscillating field amplitude (rf power) proves that the observed Rabi excitations are driven by the rf pulse (Figure S16). Moreover, for the small oscillating field **B**_1_ exploited, the oscillation’s damping factor λ is shorter than the ^*n*^*T*_2_ measured on the same transition, and it is larger for higher **B**_1_ values (Figure S16). This behavior proves that the decay of the rf-driven oscillation is dominated by the inhomogeneities of the oscillating field. It is, however, worth stressing that the effective rf field **B**_1_ at the nucleus is also enhanced by the hyperfine coupling, which is large and anisotropic and can further amplify inhomogeneities.

Nevertheless, for all of the rf power probed, the damping λ caused by pulse inhomogeneities is considerably longer than the time needed to implement a π nutation of the nuclear spin system, which is of the order of magnitude of hundreds of nanoseconds (Figure 9). This substantial difference in time scales allows implementation of several manipulations of the nuclear spin system, i.e., several 2π nutations (Figure 9) before sizeable coherence loss.

The ability to address single Δ*m*_*I*_ = ±1 nuclear transitions in **1**, driving coherent and monochromatic Rabi oscillations, is confirmed by the simulation of the nuclear system time evolution when addressed by an rf pulse, resonant to a specific nuclear transition. These simulations are based on the numerical solution of the Lindblad equation for the system density matrix ρ, assuming the Hamiltonian in eq 6 (see the Experimental Section for details). The simulations reported in Figure 9c,d show the variation in nuclear level populations when the system is targeted by an rf pulse **B**_1_ resonant with the targeted transitions (S1 and L1, respectively) in the same experimental conditions (Figure 9a,b) and including nuclear spin dephasing. On the one hand, the calculated Rabi frequencies match the experimental ones (Ω_S1_^Ex^ = 1.16 MHz, Ω_L1_^Ex^ = 1.43 MHz, Ω_S1_^Th^ = 1.09 MHz, Ω_L1_^Th^ = 1.22 MHz), within the experimental accuracy. On the other, it is immediately evident that the measured Rabi oscillations decay much faster than our calculations, confirming that the observed damping is dominated by inhomogeneities of the applied field. Most importantly, these results prove that only the populations of the targeted levels undergo a significant change, whereas the other nuclear states are unaffected by these pulses. This is confirmed by the fidelity calculated for a π rotation, which is above 99.99% for both S1 and L1 (Figure 9c,d). Moreover, our simulations show that, by optimizing the experimental conditions, it is also possible to address subsequent nuclear spin transitions (Figures S17 and S18). The individual addressability of the nuclear transitions, without affecting other nuclear states, fulfills an important requirement for the implementation of **1** as a coupled qubit–qudit unit for quantum logic operations,^28^ e.g., a qubit with embedded quantum-error correction.^49^ In addition, given the rather large hyperfine coupling *A*_*z*_ ≈ 500 MHz ≈ 25 mK, initialization of the system can be achieved by cooling. Indeed, state-of-the-art dilution refrigerators can cool the system below 5 mK.^78^ To further increase the purity of the initial state, one could also exploit the coupling to the electronic spin via algorithmic cooling methods, see, e.g., ref (79).

Being **1** a coupled qubit–qudit system we can also implement conditional dynamics depending on the state of the electronic qubit by addressing transitions between levels belonging to a given electronic spin manifold (*m*_S_ = ±1/2). Thus, complex **1** can be used to implement the general ideas developed in refs (49) and (64). In particular, the eight nuclear levels of the ^51^V nuclear spin can encode a protected logical unit, while the electronic spin 1/2 coupled by hyperfine interaction provides an ancilla to detect dephasing errors. Radiofrequency pulses addressing separately each of the seven Δ*m*_I_ = ±1 nuclear spin gaps are used to (i) encode the protected logical state |0_L_⟩ and |1_L_⟩ into proper superpositions of |*m*_I_⟩ states, (ii) manipulate these logical states, (iii) decode these states for error detection, and (iv) perform recovery operations after an error has been detected. Error detection is a crucial step of any quantum-error correction algorithm. The scheme developed in refs (49) and (64) allows us to associate different errors on a generic superposition α|0_L_⟩ + β|1_L_⟩ with different superpositions of two |*m*_I_⟩ states, namely, α|*m*_I_^′^⟩ + β|*m*_I_^″^⟩. Thanks to the sizable hyperfine interaction, we can then induce a selective excitation of the electronic spin ancilla using two microwave pulses, resonant with the two hyperfine lines corresponding to |*m*_I_^′^⟩ and |*m*_I_^″^⟩, i.e., to the error we want to probe. A measurement of the state of the electronic spin without collapsing the nuclear superposition ensures the detection of error without destruction of the quantum information contained in the coefficients α and β. Hence, the individual addressability of both nuclear and electronic spin excitations (depending on the state of the nuclear spin) is an essential requirement for the implementation of the scheme. Spectral separation of the different hyperfine lines (needed to separately detect different dephasing errors) is clearly shown in the EPR spectra of Figure 2 and S5.

## Conclusions

We have demonstrated that the combination of electronic and nuclear magnetic resonance techniques (EPR and NMR) allows obtaining an accurate description of this organometallic complex. This holistic magnetic resonance multifrequency approach provided complementary but not redundant information on the [V(Cp)_2_Cl_2_] spin Hamiltonian and allowed us to evaluate and compare its electronic and nuclear spin dephasing times.

The ability to coherently manipulate both the nuclear and electronic spin multilevel structures is demonstrated by nutation experiments in which Rabi oscillations can be driven by either microwave or radiofrequency excitation pulses, resonant to specific system transitions.

Even if the simultaneous coherent manipulation of nuclear and electronic spins will require the development of an *ad hoc* experimental setup, the achieved degree of control of this multilevel system, together with the remarkably long coherence times, places this vanadium(IV) organometallic complex among the promising coupled qubit–qudit candidates for quantum applications.

## Experimental Section

### General Remarks

Compounds **1** and **2** were commercially available and were purchased and used as received from Sigma-Aldrich.

### Preparation of Crystalline Dispersions

Crystalline dispersions and single crystals were obtained by dissolving weighed amounts of the starting materials in 10, 1, and 0.1% nominal concentrations in CH_2_Cl_2,_ followed by slow evaporation of the solvent in a N_2_ controlled atmosphere.

### Characterization

The effective concentrations of vanadium(IV) in the titanium(IV)-based crystalline dispersions **3a–3c** were determined through an inductively coupled Plasma Perkin Elmer Optima 2000 OES DV spectrophotometer.

### Powder X-ray Crystallography

Wide-angle powder X-ray diffraction (PXRD) patterns on polycrystalline samples were recorded on a Bruker New D8 Advance DAVINCI diffractometer in a θ–θ configuration equipped with a linear detector. The scans were collected within the range 5–40° (2θ) using Cu Kα radiation (λ = 1.540 Å). Simulated patterns were generated from the atomic coordinates of the single-crystal structure solutions using Mercury CSD 3.5 software (copyright CCDC, http://www.ccdc.cam.ac.uk/mercury/) using a full width at half-maximum (FWHM) of 0.10 and a 2θ step of 0.025.

### Electron Paramagnetic Resonance

CW X-Band EPR spectra of all samples were recorded on a Bruker Elexsys E500 spectrometer equipped with an SHQ cavity (ν ≅ 9.405 GHz). To perform single-crystal measurements, a crystal of **3c** glued on an acetate sheet was indexed using an Oxford Diffraction XCalibur diffractometer (Cu Kα (λ = 1.54056 Å) radiation and graphite monochromator) equipped with a CCD detector. This was then transferred to the polyethylene rod sample holder: its orientation with respect to the magnetic field was controlled by a digital programmable goniometer (ER218PG1, Bruker BioSpin). Low-temperature measurements were obtained using an Oxford Instruments ESR900 continuous flow helium cryostat. Continuous-wave Q-band (ν ≈ 33.8 GHz) EPR measurements were performed on a Bruker Elexsys E580 spectrometer equipped with an EN 5107D2 resonator (Bruker). The temperature was controlled with an Oxford Instruments CF935 helium flow cryostat and an ITC503 temperature controller. Experimental conditions were as follows: microwave power = 0.2 mW, conversion time = 40.96 ms, time constant = 10.24 ms, modulation frequency = 50 kHz, and modulation amplitude = 0.1 mT.

Pulsed EPR measurements were carried out with a Bruker Elexsys E580 at X-band (ν ≅ 9.70 GHz) equipped with a flexline dielectric ring ENDOR resonator (Bruker EN 4118X-MD4). Temperatures between 4.5 and 250 K were obtained with an Oxford Instruments CF935 continuous flow helium cryostat. EDFS EPR spectra were recorded using the Hahn Echo pulse sequence (π/2−τ–π–τ–echo) with fixed interpulse delay time τ = 200 ns, *t*_π/2_ = 16 ns, and *t*_π_ = 32 ns. Phase memory times were measured both by the Hahn Echo sequence upon increasing the interpulse delay τ starting from τ = 98 ns. Typical pulse lengths were *t*_π/2_ = 16 ns and t_π_ = 32 ns. Spin–lattice-relaxation times were measured using the standard inversion recovery sequence (π–*t*_d_–π/2−τ–π–τ–echo), with π/2 = 16 ns. Electron spin Rabi nutations were measured with the sequence *t*_p_–*t*_d_–π/2−τ– π–τ–echo with a nutation pulse (*t*_p_) of variable length, followed by a Hahn echo sequence

ENDOR ^1^H hyperfine couplings were detected using the Mims^80^ pulse sequence π/2−τ–π/2−π_RF_–π/2−τ–echo with π/2 = 16 ns, π_RF_ = 14 μs and additional waiting times of 1 μs were used before and after the π_RF_ pulse. To avoid blind spots, ten spectra with τ values from 100 to 260 ns were recorded and summed together. Mims ENDOR spectra were recorded in a 14 MHz window centered at the ^1^H Larmor frequency, with a 0.027 MHz spectral resolution.

Spectral simulations were performed using EasySpin^72^ for powder distribution of microcrystallites. We note that a proper simulation of the relative intensity of the lines of the EDFS spectrum required to assume some preferential orientation of the crystallites. Such a choice might, in principle, just mimic the effect of relaxation anisotropy or hyperfine modulation effects (ESEEM) so that some components of the spectrum are filtered away in echo detection.

### Nuclear Magnetic Resonance

The NMR spin-echo measurements were carried out by means of a home-built broadband, phase-coherent, NMR spectrometer optimized for the study of magnetic materials, named “HyReSpect.”^81^ The sample environment consists of a Maglab EXA (Oxford Instruments) 0–9 T superconducting magnet equipped with a helium flow insert, ensuring stable temperature control. The radiofrequency pulses were amplified by a linear pulse power amplifier and fed into the NMR probehead. The probehead, hosted by the cryostat, consists of an LC resonant circuit, made of a small coil (∼300 nH depending on the target frequencies), which is coiled around a three-dimensional (3D) printed nonmagnetic plastic crystal holder, and a Voltronics NTNM-120 variable capacitor, providing a wide tuning range. The employment of a variable capacitor allowed automated frequency scans with an automatic probe tuning system. NMR spectra were recorded by exciting and detecting spin echoes over frequency spans properly tuned for each magnetic field direction, at discrete 0.1 MHz for a single crystal of **3b**. The spin echoes were excited by a (2π/3−τ–2π/3) pulse sequence,^82^ consisting of two equal rf pulses (*t*_pulse_ = *t*_echo_ = 0.1–0.2 μs), that has been optimized to achieve maximum resonance signals, and a delay τ ca. 4–10 μs, kept as short as possible (limited by the dead time of the apparatus). The same Hahn sequence was employed to measure the nuclear phase memory times, increasing the delay τ between the exciting and refocusing pulses to reconstruct the single exponential decay. Meanwhile, the pulse sequence exploited for the excitation and detection of nuclear Rabi oscillations was composed of the first pulse of variable length θ(*t*), inducing a rotation of a generic angle θ of the spin system, separated by a properly chosen delay τ, from a refocusing π-pulse. The overall excitation bandwidth is in the order of ±1 MHz (sufficiently narrow to permit single transition addressability, as demonstrated above) and is defined by the product of the exciting rf field B_1_, the finite spectrometer bandwidth, and the gain factor Q of the LC probe.

### Lindblad Time Evolution Simulation

The time evolution of the system subjected to an exciting rf pulse has been simulated by numerical solution of the Lindblad equation for the system density matrix, with the first term representing a coherent evolution and a second term that defines the nuclear spin dephasing: 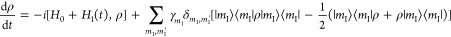
as previously reported in the literature for similar complexes.^64,83^ Here, *H*_0_ stands for the static Hamiltonian, while *H*_1_(*t*) represents the time-dependent exciting pulse. The nuclear dephasing rate was instead defined for each transition *m*_*I*_ → *m*_*I*_^′^ as . The Hamiltonian experimentally defined by EPR and NMR and the measured *T*_2_’s were used as starting points for simulating the system evolution under the perturbation of the tuned rf pulse.

## Supporting Information Available

Additional figures describing the molecular structure and packing; powder X-ray diffraction spectra and crystal morphology (Figures S1–S4); additional EPR characterization (Figures S5–S7); details of the single-crystal NMR for Spin Hamiltonian characterization (Figures S8 and S9); angular dependence of the EPR resonance fields in *xz-* and *zy-*planes (Figure S10); decay trace for ^e^*T*_1_ and ^e^*T*_m_ determination at variable temperatures (Figures S11 and S12); ENDOR ^1^H spectra of **3c** (Figure S13); and additional NMR characterization of the nuclear dynamics (Figures S14–S18) (PDF)
