# Supplementary material for: Radiofrequency to Microwave Coherent Manipulation of an Organometallic Electronic Spin Qubit Coupled to a Nuclear Qudit
Source: Inorg Chem. 2021 Jul 15;60(15):11273–86. doi: 10.1021/acs.inorgchem.1c01267 (PMC8389802; doi:10.1021/acs.inorgchem.1c01267)
Supplement: Supplementary file 1 — ic1c01267_si_001.pdf [file ic1c01267_si_001.pdf]

Radiofrequency to Microwave Coherent Manipulation of an Organometallic Electronic Spin Qubit coupled to a Nuclear Qudit

Matteo Atzori,[†,‡] Elena Garlatti,[||] Giuseppe Allodi,[||] Simone Chicco,[||] Alessandro Chiesa,[||] Andrea Albino,[†] Roberto De Renzi,[||] Enrico Salvadori,[§] Mario Chiesa,[§] Stefano Carretta[||] and Lorenzo Sorace[†,*]

[†] Dipartimento di Chimica "Ugo Schiff" e UdR INSTM, Università degli Studi di Firenze, Via della Lastruccia 3, I-50019 Sesto Fiorentino (Firenze), Italy

[‡] Laboratoire National des Champs Magnétiques Intenses (LNCMI), Univ. Grenoble Alpes, INSA Toulouse, Univ. Toulouse Paul Sabatier, EMFL, CNRS, F-38043 Grenoble, France

[||] Università di Parma, Dipartimento di "Scienze Matematiche, Fisiche e Informatiche" e UdR INSTM, I-43124 Parma, Italy

[§] Dipartimento di Chimica e NIS Centre, Università di Torino, Via P. Giuria 7, I-10125 Torino, Italy

* e-mail: lorenzo.sorace@unifi.it

SUPPLEMENTARY INFORMATION

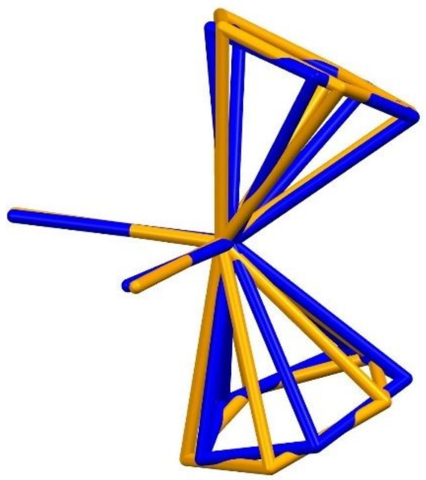

Figure S1. Overlay of the molecular structure of [$V(Cp)_2Cl_2$] (orange, monoclinic *P21/c* space group) and of [$Ti(Cp)_2Cl_2$] (blue, triclinic *P−1* space group).

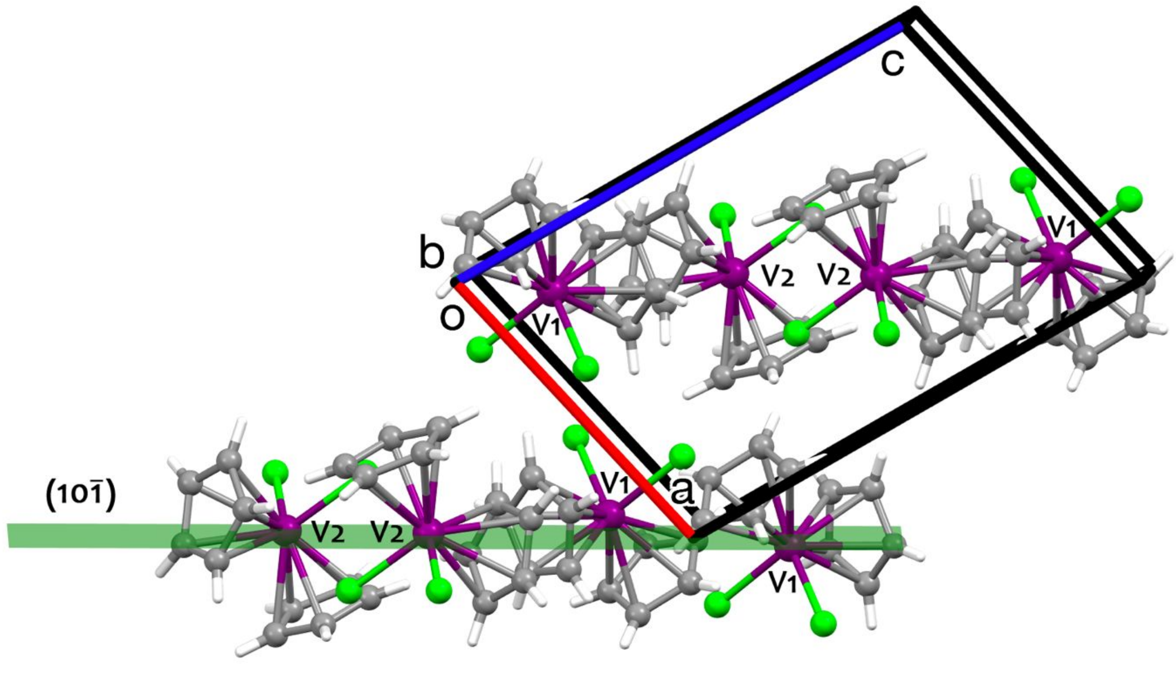

Figure S2. View of the crystal structure of **1** along the *b* crystallographic axis with the crystal packing of the molecule along the (10-1) plane highlighted.

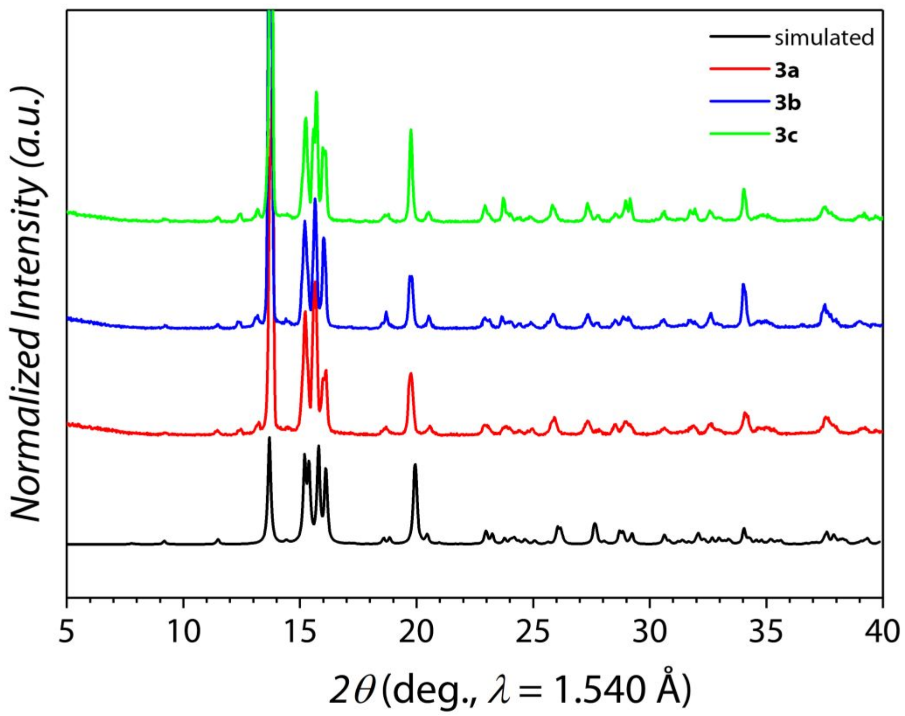

Figure S3. Comparison between experimental (see legend) and simulated (black line, triclinic space group crystal structure of **2**) PXRD patterns (5–40°, 2θ) for **3a-3c**.

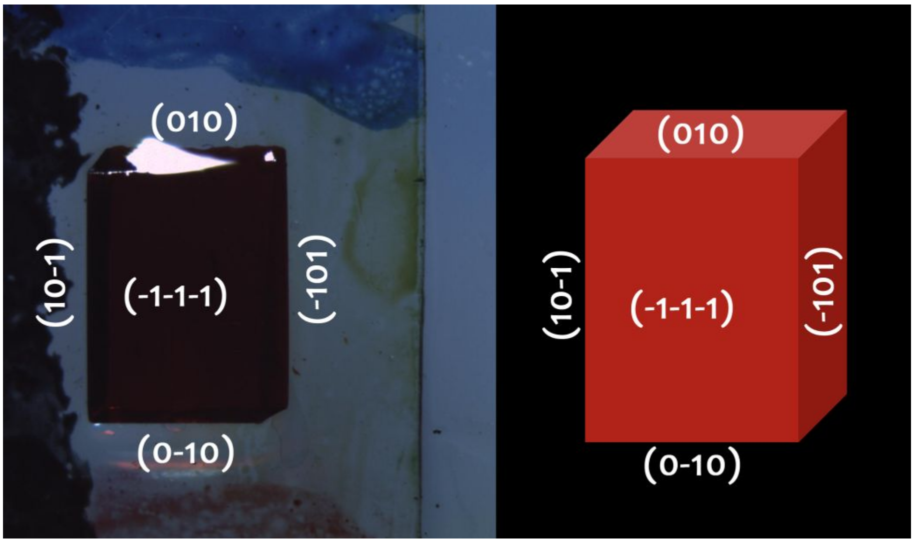

Figure S4. Optical image (left) and schematic representation (right) of a single crystal of **3c** used for EPR and NMR measurements with crystal face indexing.

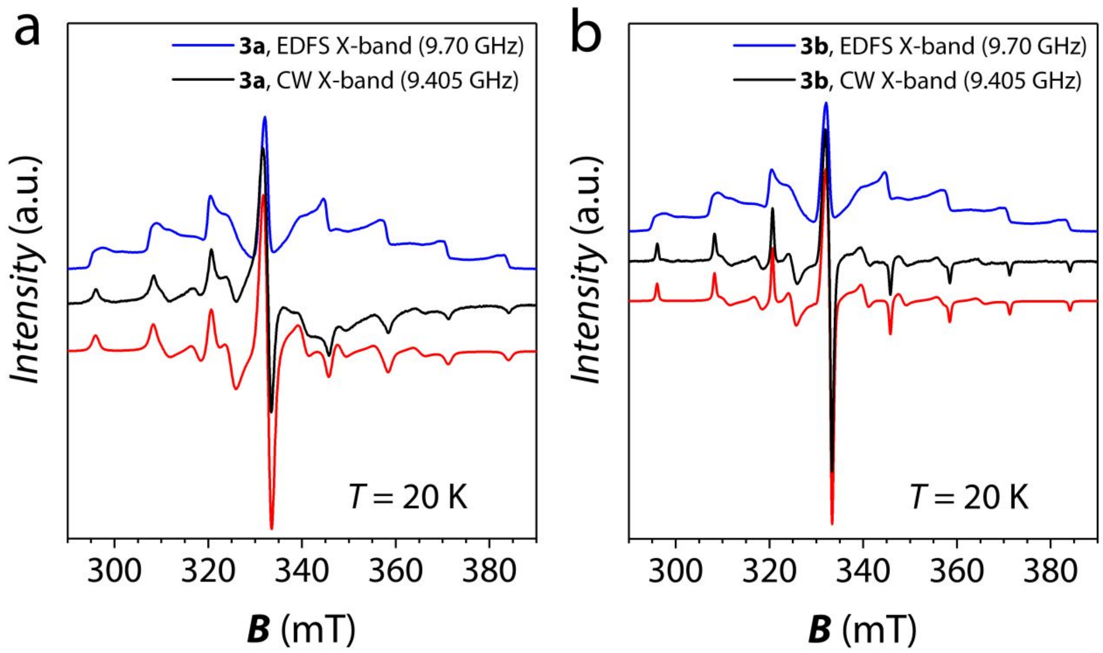

Figure S5. Experimental EDFS (blue line) and CW (black lines) X-band EPR spectra for **3a** (a) and **3b** (b) recorded at $T = 20$ K. To ease the comparison with CW data, EDFS data were translated to account for frequency difference. The spectral simulations corresponding to the spin Hamiltonian parameters reported in the text are shown in red. In the experimental spectrum of **3a** a broad band is clearly observed probably due to a clustering of the paramagnetic components that has not been included in the simulation (red line) and does not contribute to the EDFS spectrum (blue line).

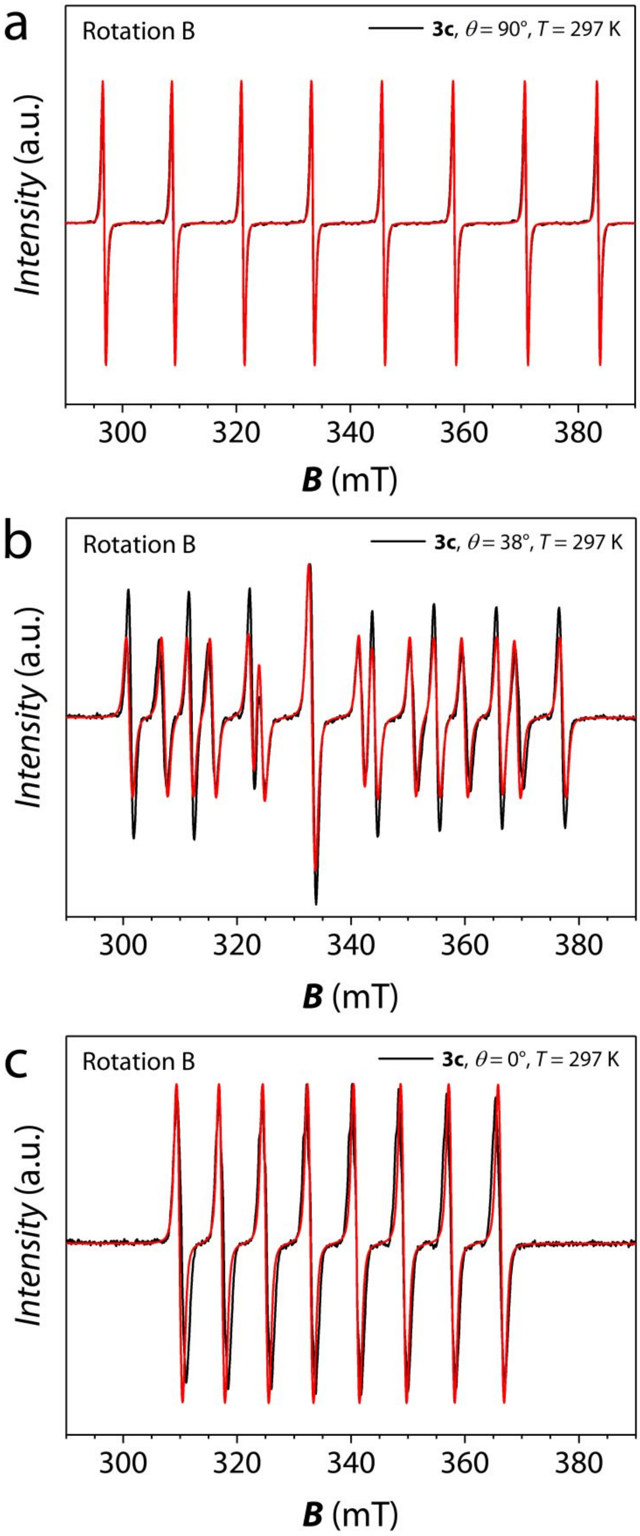

Figure S6. Experimental (black line) and simulated (red lines) X-band EPR spectra for different orientations $\theta = 90°$ (a), 38° (b), 0° (c) of a single crystal of **3c** recorded through rotation B. Spectra at $\theta = 90°$ and 0° show no difference between the two molecular species while at $\theta = 38°$ the EPR signal for the two differently oriented species are clearly observed.

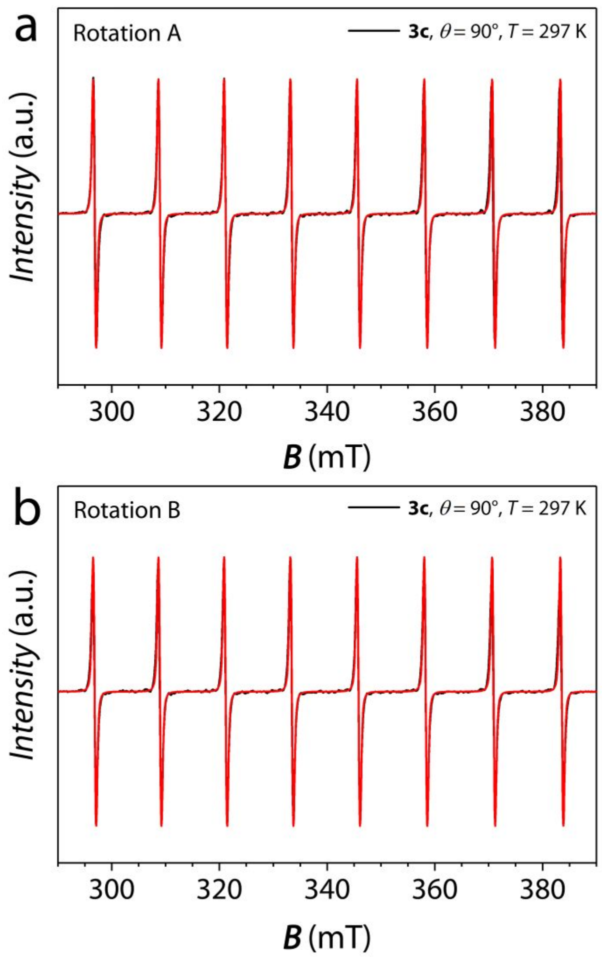

Figure S7. Experimental (black line) and simulated (red lines) X-band EPR spectra for the same orientation (θ= 90°) of the crystal during rotation A (a) and rotation B (b). The extension of the spectra clearly shows that the same hyperfine coupling along *Z*.

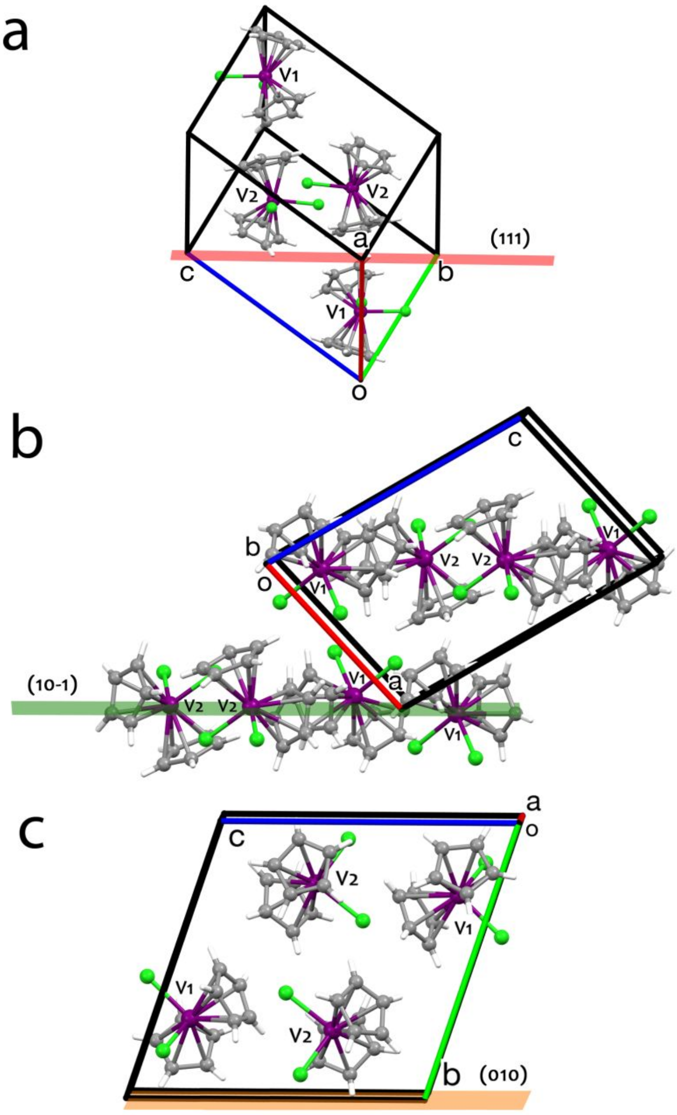

Figure S8. Lateral view of the molecular and crystal packing of the crystalline dispersions **3a-3c** (triclinic space group *P–1*) lying on face (111) (a), face (-101) (b), and face (010) ($\boldsymbol{B}_L$ configuration) (c).

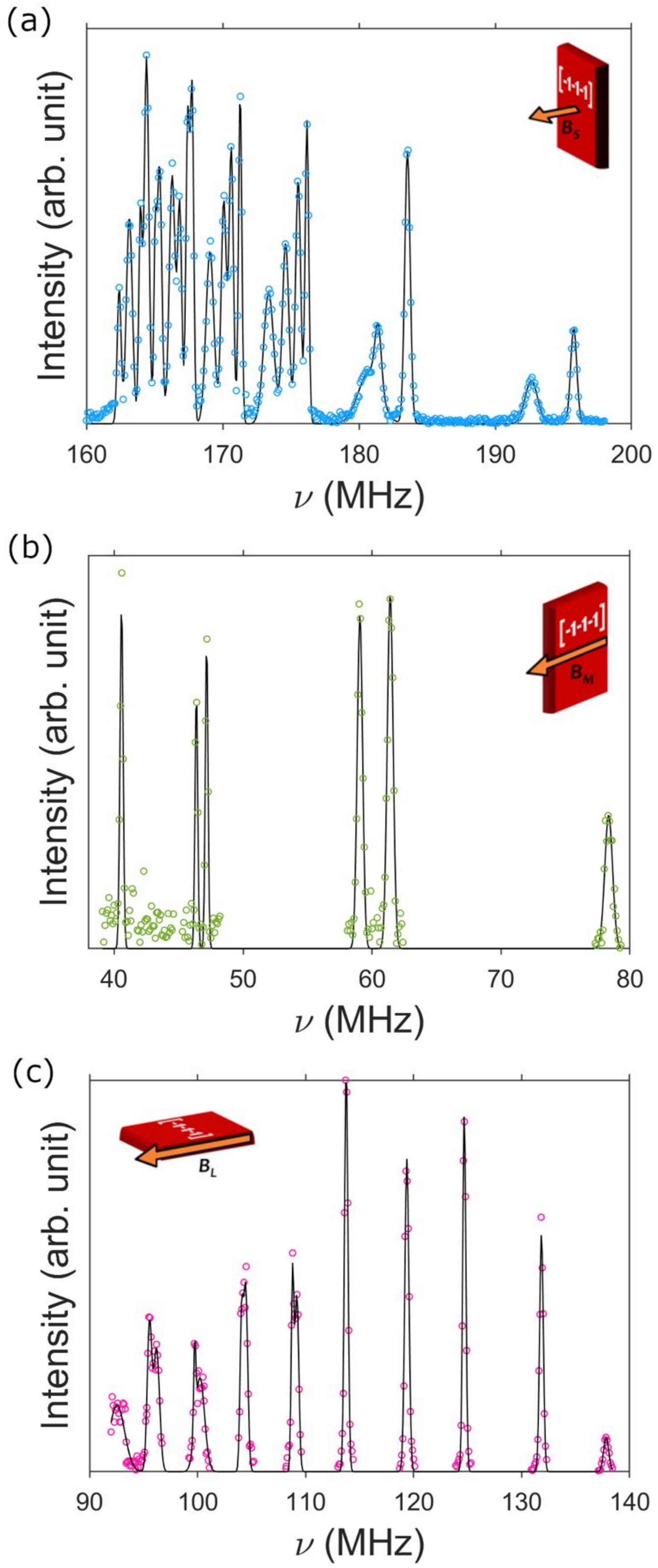

Figure S9. Experimental NMR spectra (dots) at T = 4 K for a fixed static field $\boldsymbol{B}$ = 0.2 T, applied along all the direction indexed for the single crystal (inset) $\boldsymbol{B}_S$ (a), $\boldsymbol{B}_M$ (b) and $\boldsymbol{B}_L$ (c). Solid black lines represent the fitting with gaussian lines shapes for each transition.

Table S1 Nominal crystallographic directions and refined orientations of the applied magnetic field with respect to the *xyz* magnetic tensor frames in term of the azimuthal and polar angles θ and ϕ, for each configuration.

Probed directions	*Crystallographic directions*	*Azimuthal Angle* θ *(deg.,°)*	*Polar Angle* ϕ *(deg.,°)*
$\boldsymbol{B}_S$	[111]–[-1-1-1]	78(1)	33.9(2)
$\boldsymbol{B}_M$	[-101]–[10-1]	119(1)	120.0(2)
$\boldsymbol{B}_L$	[1 2 1]–[-1 -2 -1]	29(1)	143.0(2)

Table S2 Director cosines of the principal directions of **g** and **A** tensors in the *ab'c** cartesian reference system.

	**Molecule 1** (EPR)			**Molecule 2** (EPR)			**Molecule 1** (NMR)			**Molecule 2** (NMR)		
	a	*b'*	*c**	*a*	*b′*	*c**	*a*	*b'*	*c**	*a*	*b′*	*c**
x	0.3833	-0.8069	0.4495	0.4404	-0.7692	0.4630	0.3762	-0.8063	0.4552	0.5385	-0.7172	0.4432
y	0.2181	-0.3967	-0.8917	0.5585	-0.1723	-0.8114	0.2528	-0.3835	-0.8882	0.5104	-0.1410	-0.8483
z	0.8980	0.4393	0.0241	0.7036	0.6165	0.3535	0.891	0.449	0.059	0.671	0.683	0.29

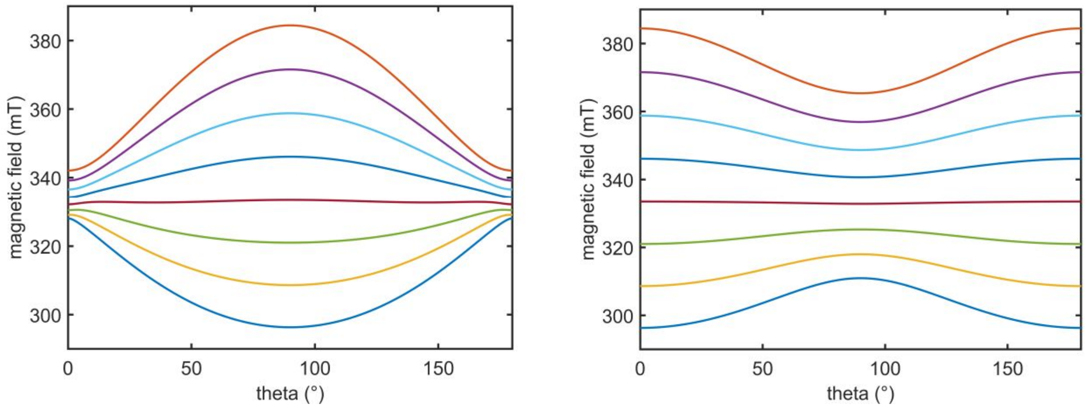

Figure S10 Calculated angular dependence of the EPR resonance fields in the *xz* (left) and *zy* (right) planes, evidencing the largely isotropic nature of the m_I=-1/2 transition.

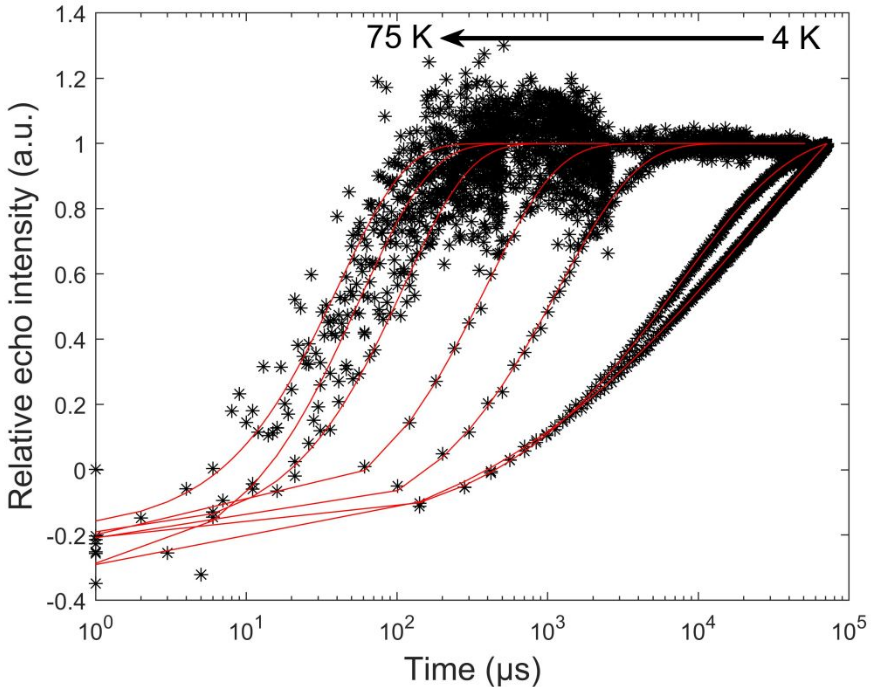

Figure S11 Pulsed EPR inversion recovery traces recorded for **3c** recorded at different temperatures and best fit curves based on Eq. (2) of main text.

Table S3. Best-fit parameters of the models used to simulate the temperature dependence of ${}^{e}T_1$ extracted by pulsed EPR experiments.

	Parameters for Equation (3)			Parameters for Equation (4)		
**Compound**	a (μs^{-1} K^{-1})	b (μs^{-1} K^{-n})	n	c (μs^{-1} K^{-1})	d (μs^{-1})	$\hbar\omega$ (cm^{-1})
**3a**	$4.4(1) \cdot 10^{-4}$	$5(1) \cdot 10^{-6}$	2.4(1)	-	-	-
**3b**	$7.5(1) \cdot 10^{-5}$	$8(1) \cdot 10^{-8}$	3.2(1)	-	-	-
**3c**	$3.9(1) \cdot 10^{-6}$	$4(1) \cdot 10^{-11}$	4.8(1)	$1.8(1) \cdot 10^{-6}$	0.36(1)	120(10)

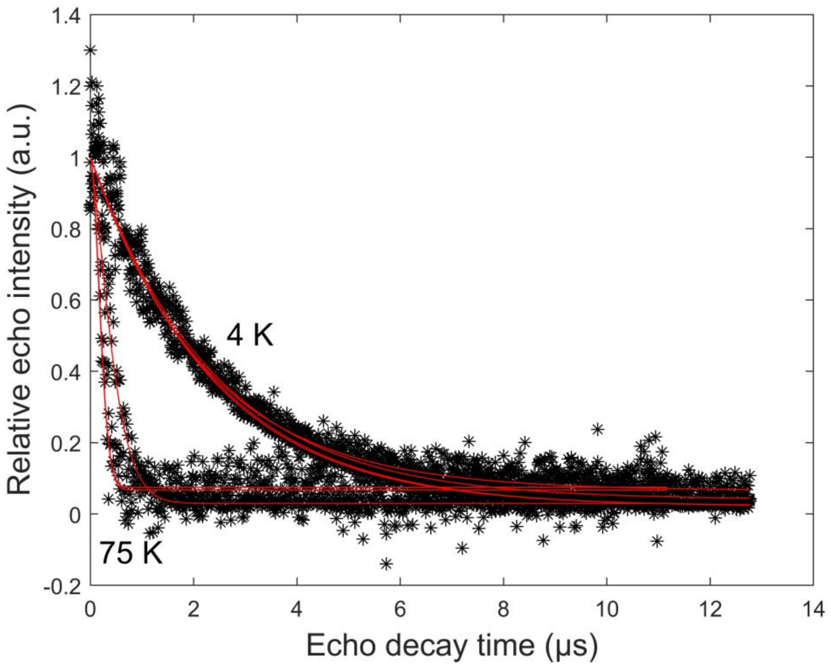

Figure S12 Pulsed EPR spin-echo decay traces of **3c** measured at different temperatures and best-fit curves obtained based on Eq. (5), main text.

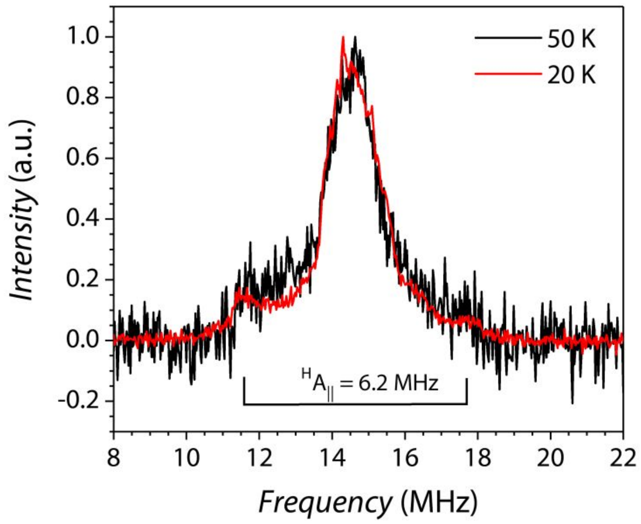

Figure S13. Q-band Mims ENDOR spectra recorded at 20 K and 50 K (see legend) on a microcrystalline sample of **3c**. The extracted value of $^{H}A_{||}$ remains constant for the two selected temperatures.

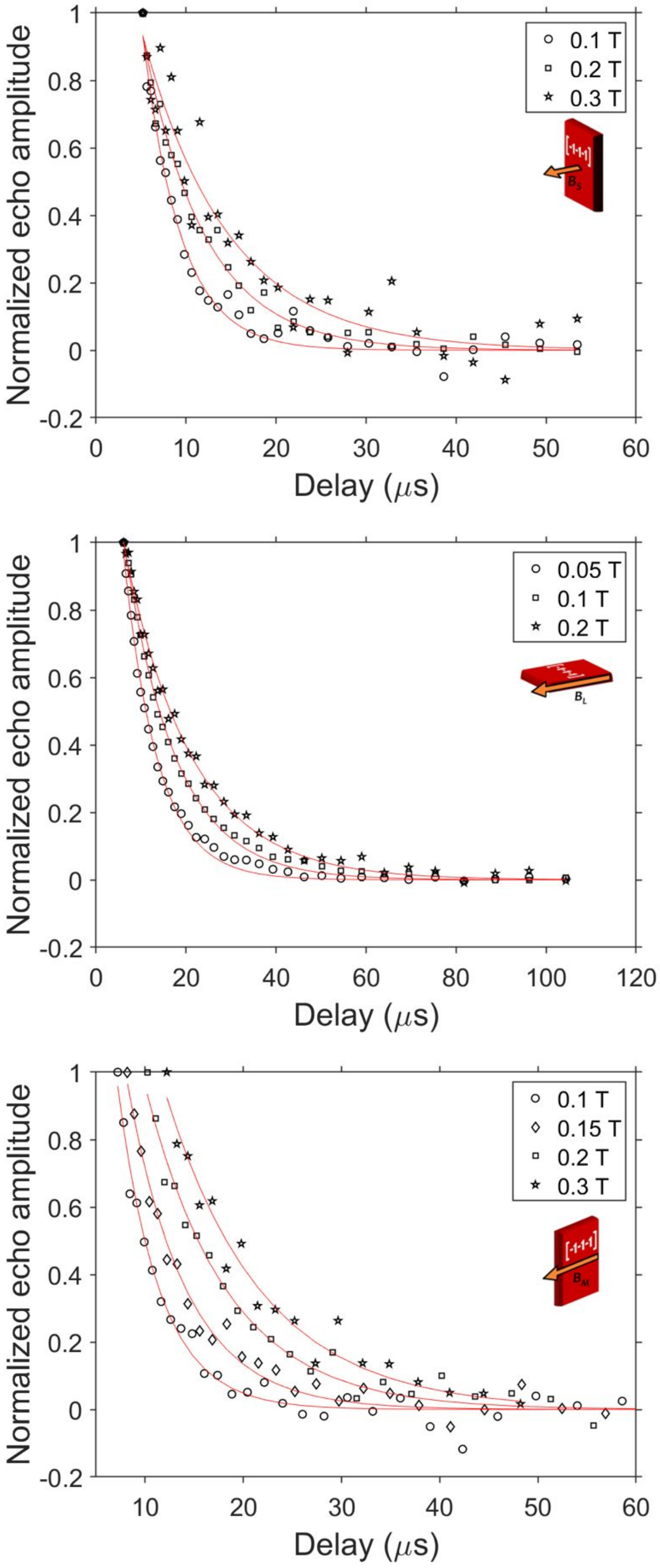

Figure S14. Echo decay experiments, showing that the transverse magnetization relaxation follows a single exponential law as a function of the delay between exciting and refocusing pulses. Dots represent the echoes intensities, while lines represent the single exponential fitting curves.

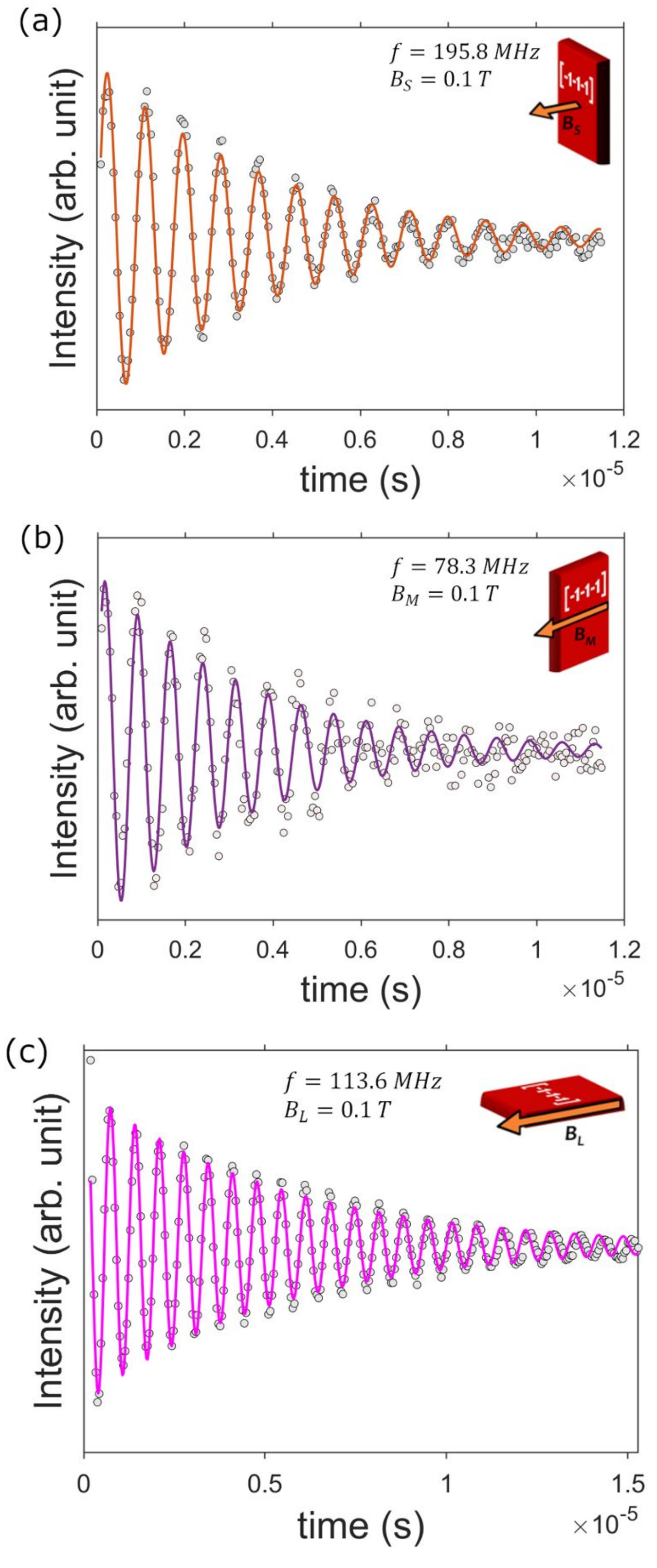

Figure S15. Nuclear Rabi oscillations driven by a rf pulse for the static field applied along $\boldsymbol{B}_S$ (a), $\boldsymbol{B}_M$ (b) and $\boldsymbol{B}_L$ (c). Dots are experimental data, while lines represent the fitting function (exponentially damped sinewave) described in the main text. See insets for details on the excited transitions frequencies (*f*) and field intensities and directions ($\boldsymbol{B}_S$, $\boldsymbol{B}_M$, $\boldsymbol{B}_L$)

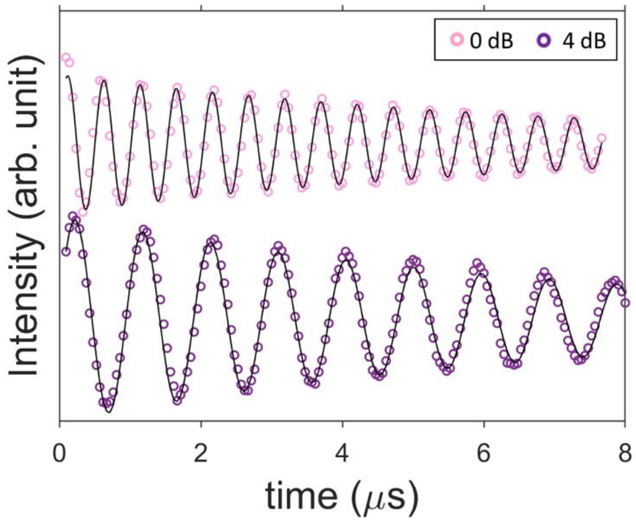

Figure S16. Rabi Oscillation induced on the same transition (f = 110.8 MHz) at fixed static magnetic field B_0 = 0.2 T (long side $\boldsymbol{B}_L$) for different rf pulse attenuations, showing the variation of Rabi frequency $^n\Omega_R$ as a function of the pulse intensities.

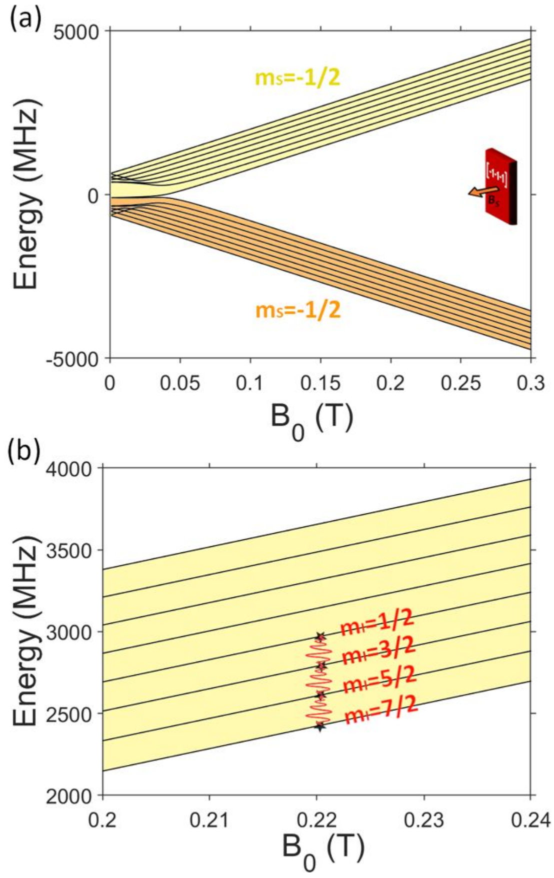

Figure S17. (a) Nuclear levels in both the electronic spin multiplets m_S = ±1/2, for the static field $\boldsymbol{B}_S$ applied along the crystal shortest side (see inset). (b) Close-up of the nuclear spin-states of the m_S = +1/2 manifold, whose populations are manipulated by properly tuned rf pulses (red marks) as in the simulations of Figure S18. The m_I labels are correct to 0.5-1% in the external dc field considered.

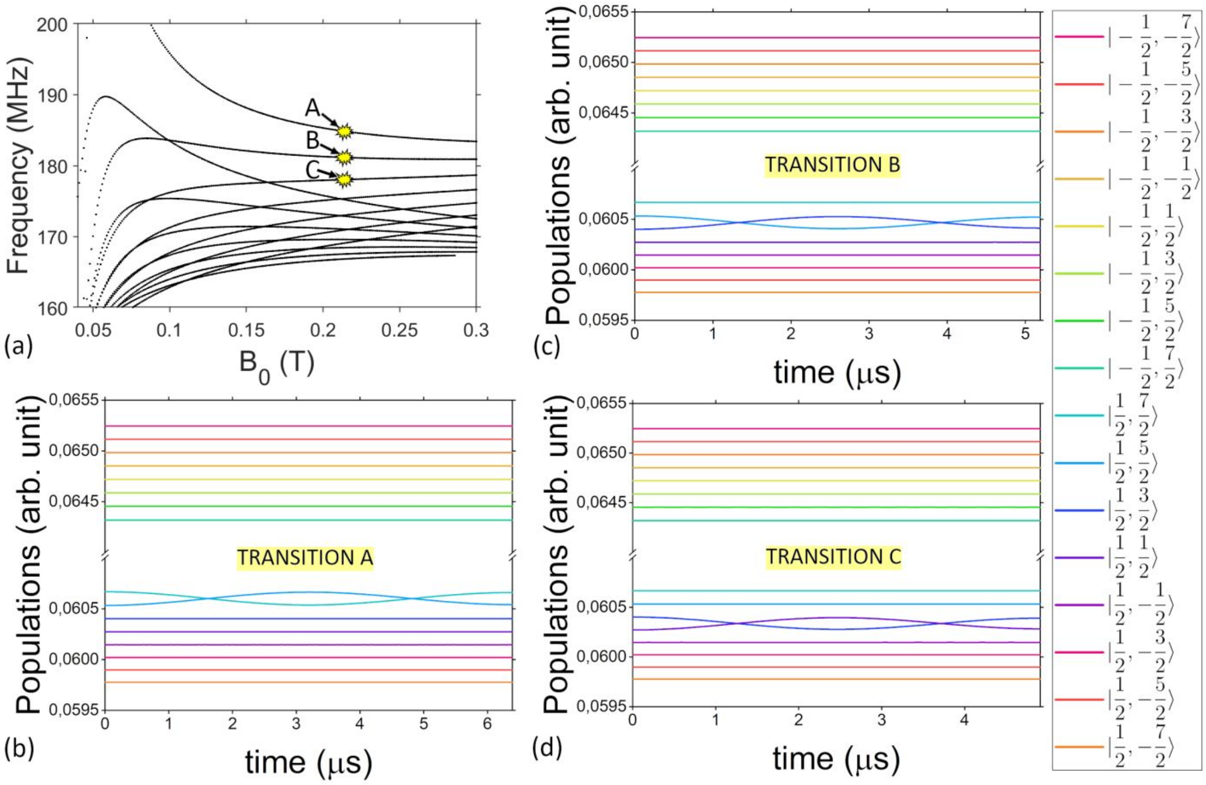

Figure S18. (a) Diagram of the calculated spectral position of the target transitions **A**, **B**, **C**. (b, c, d) Simulation of coherent and monochromatic Rabi oscillations induced between the four subsequent nuclear states (b) $m_I = 7/2 \rightarrow 5/2$, (c) $m_I = 5/2 \rightarrow 3/2$, (d) $m_I = 3/2 \rightarrow 1/2$, shown in figure S15, with the static magnetic field applied along the $\boldsymbol{B_S}$ direction (transitions **A**, **B**, **C** respectively). The population variation concerns only the targeted levels for properly tuned static and oscillating fields ($\boldsymbol{B_S}$, $\boldsymbol{B_1}$), with negligible leakage to other nuclear states. $\boldsymbol{B_S} = 0.22$ T displays the widest spectral separation between transitions, while $\boldsymbol{B_1}$ = 2 G grants monochromatic oscillation, with a period still much shorter than T_2. The m_I labels are correct to 0.5-1% in the external dc field considered.

Effect of electron nuclear mixing on $^nT_2$ and Rabi frequency

Nuclear Spin decoherence and Rabi frequencies are both influenced by the electro-nuclear mixing. Indeed, in the examined magnetic field range, the mixing between electronic and nuclear spin wave-function provides the dominant contribution for both processes. On the one hand, the Rabi frequency is proportional to matrix elements of transverse spin operators $g_I\mu_N I_x + g_x\mu_B S_x$ between pairs of eigenstates, while $^nT_2$ is proportional to the difference of expectation values of $g_I\mu_N I_z + g_z\mu_B S_z$ between the pair of examined states. In both cases, since $g_x\mu_B \gg g_I\mu_N$, the small electronic mixing of the wave-function ($\propto 1/B$) is responsible of almost the entire effect. Hence, the nuclear $^nT_2$ acquires some of the electron spin decoherence.

We expect that for magnetic fields fulfilling $g\mu_B B \gg A_{x,y}$, the Rabi frequency to be proportional to $1/B$ (i.e. to the off-diagonal element of S_x), whereas the dephasing rate scales as $1/B^2$, because it involves diagonal matrix elements of S_z. Electron spin coherence is also influenced by other factors, such as polarization of the surrounding electronic spins, an effect which also increases by increasing B, thus enhancing the electronic and (indirectly) also the nuclear ${}^{n}T_2$.
